# Taxonomic identification and antagonistic activity of *Streptomyces luomodiensis* sp. nov. against phytopathogenic fungi

**DOI:** 10.3389/fmicb.2024.1402653

**Published:** 2024-05-27

**Authors:** Dengfeng Qi, Qiao Liu, Liangping Zou, Miaoyi Zhang, Kai Li, Yankun Zhao, Yufeng Chen, Junting Feng, Dengbo Zhou, Yongzan Wei, Wei Wang, Lu Zhang, Jianghui Xie

**Affiliations:** ^1^National Key Laboratory of Biological Breeding of Tropical Crops, Institute of Tropical Bioscience and Biotechnology, Chinese Academy of Tropical Agricultural Sciences, Haikou, China; ^2^Ministry of Education Key Laboratory for Ecology of Tropical Islands, Key Laboratory of Tropical Animal and Plant Ecology of Hainan Province, College of Life Sciences, Hainan Normal University, Haikou, China

**Keywords:** *Streptomyces*, novel species, antifungal activity, taxonomic identification, banana *Fusarium* disease, antagonistic mechanism

## Abstract

Banana wilt caused by *Fusarium oxysporum* f. sp. *cubense* tropical race 4 (Foc TR4) is a devastating fungal disease. Biocontrol strategies hold immense potential for inhibiting the spread of Foc TR4. Here, 30 actinobacteria were isolated from soils and screened for their antagonistic activity against Foc TR4. Strain SCA4-21^T^ was selected due to its strongest antagonistic activity against Foc TR4. Strain SCA4-21^T^ also exhibited strong antagonistic activity against the other eight phytopathogenic fungi. The strain was identified as the genus *Streptomyces* according to its physiological, biochemical, and phenotypic characteristics. The phylogenetic trees of 16S rRNA sequences demonstrated that strain SCA4-21^T^ formed a subclade with *S. iranensis* HM 35^T^ and/or *S. rapamycinicus* NRRL B-5491^T^ with low bootstrap values. Considering that 16S rRNAs did not provide sufficient resolution for species-level identification, the whole genome of strain SCA4-21^T^ was sequenced. Multilocus sequence analysis (MLSA) based on five housekeeping gene alleles (*atpD*, *gyrB*, *recA*, *rpoB*, and *trpB*) revealed that strain SCA4-21^T^ clustered into *S. hygroscopicus* subsp. *hygroscopicus* NBRC 13472^T^ with 100% of bootstrap value. The analysis of the genome-based phylogeny also approved the results. Average nucleotide identity (ANI) and digital DNA–DNA hybridization (dDDH) were 91.26 and 44.30%, respectively, with values below the respective species level threshold of 95 and 70%. Hence, strain SCA 4–21^T^ represented a novel species within the genus *Streptomyces*, named *Streptomyces luomodiensis* sp. nov. The type strain is SCA4-21^T^ (=GDMCC4.340^T^ = JCM36555^T^). By the CAZymes analysis, 348 carbohydrate-active enzymes (CAZymes) were detected, including 15 chitinases and eight β-1,3-glucanases. The fermentation broth of strain SCA4-21^T^, exhibiting strong antagonistic activity against Foc TR4, demonstrated high activities of chitinase and β-1,3-glucanase, which might be involved in antifungal activity. Our results showed an innovative potential biocontrol agent for managing plant fungal diseases, specifically banana *fusarium* wilt.

## Introduction

Bananas (*Musa* spp.) are an important fruit and staple food crop in the tropics and subtropics (Mohandas and Ravishankar, 2016). However, the world banana industry is suffering from the serious harm of *Fusarium* wilt disease, which is caused by the soil-borne *Fusarium oxysporum* f. sp. *cubense* (Foc). Foc race 1 (Foc 1) destroyed the major cultivar of the Gros Michel banana in Central America and the Caribbean during the mid-20th century (Pérez-Vicente et al., 2014). Although the detriment of Foc 1 was overcome by the resistant Cavendish cultivars, a new strain of Foc known as tropical race 4 (Foc TR4) emerged and spread rapidly (Ploetz, 2015). Foc TR4 can infect almost all banana cultivars. It is difficult to control Foc TR4 because of its long survival ability in soil (Ploetz, 2015). Utilizing antagonistic microbes to control Foc TR4 is a promising strategy due to its environmentally friendly and highly effective advantages (Fu et al., 2017).

Actinobacteria are ubiquitously distributed in the soil and natural environment. They are considered an ideal candidate for biocontrol agents (Gonzalez-Franco and Hernandez, 2009; Bhatti et al., 2017). Actinobacteria successfully colonizing the plant rhizosphere provides a biocontrol potential for soil-borne disease (Barka et al., 2015). *Streptomyces* is the largest genus of the phylum *Actinomycetota* (Law et al., 2017). They are Gram-positive, aerobic, filamentous bacteria with approximately 70% of G + C content in their genomes (Waksman and Henrici, 1943). The traditional method of DNA–DNA hybridization allowed the classification of prokaryotes, but serious shortcomings limited its application such as a time-consuming procedure and operational feasibility (Gevers et al., 2005). Currently, the identification of *Streptomyces* primarily relies on the combination of phenotypic and genotypic characteristics. 16S rRNA sequences did not provide a sufficient resolution for the species-level identification. Average nucleotide identity (ANI) provided an efficient method to identify the level of species (Gevers et al., 2005). At the moment of writing, there are 726 *Streptomyces* species with a validly published and correct name, according to the LPSN–list of Prokaryotic names with Standing in Nomenclature (Parte et al., 2020).^1^ However, there is still a lack of *Streptomyces* with strong adaptability and high antimicrobial activity in agricultural practice.

*Streptomyces* are also massive reservoirs of bioactive substances such as antibiotics, enzymes, and siderophores (Jose et al., 2021). Moreover, they also participate in the cycling of carbon due to their ability to secrete many carbohydrate-active enzymes (CAZymes). These enzymes can facilitate the biodegradation of remains from animals, plants, and fungi (Barka et al., 2015). CAZymes are classified into six major families according to the functional characteristics (Mann et al., 2013). The members of the glycoside hydrolase family contain chitinase and β-1,3-glucanase (Aktuganov et al., 2008; Zacky and Ting, 2013). Chitinase and β-1,3-glucanase are responsible for the antifungal activity of *Streptomyces* sp. 5–4 (Yun et al., 2022) and *Streptomyces griseus* (Anitha and Rebeeth, 2009). However, the antifungal mechanism of *Streptomyces* is still unclear due to the physiological property differences of diverse members.

In this study, 30 actinobacteria were isolated from a dry-hot valley of Huili County, Sichuan Province, China. Their antagonistic activity was screened against Foc TR4. Strain SCA4-21^T^ was further investigated due to its strongest antagonistic activity against Foc TR4. The broad-spectrum antifungal activity of strain SCA4-21^T^ was initially detected against the selected eight phytopathogenic fungi. Subsequently, the taxonomic status of strain SCA4-21^T^ was identified based on its phenotypic and genotypic characteristics. To explore the antifungal mechanism of strain SCA4-21^T^, CAZymes of its genome were predicted, and the antifungal activity of the fermentation broth from strain SCA4-21^T^ was determined. Additionally, the activities of chitinase and β-1,3-glucanase in the fermentation broth from strain SCA4-21^T^ were tested.

## Materials and methods

### Isolation of actinobacteria

The soil samples were collected from the Luomodi Village in a dry-hot valley of Huili County, Sichuan Province, China. Actinobacteria were isolated using a serial dilution method on the starch-casein medium (SCA, 10 g of soluble starch, 0.3 g of casein, 2.0 g of KNO_3_, 2.0 g of NaCl, 2.0 g of K_2_HPO_4_, 0.05 g of MgSO_4_⋅7H_2_O, 0.02 g of CaCO_3_, 0.01 g of FeSO_4_⋅H_2_O, and 18 g of agar in 1 L sterile ddH_2_O, pH 7.0–7.4). The addition of potassium dichromate (50 mg/L) and nystatin (50 mg/L) was used to suppress the growth of other fungi and bacteria, respectively (Williams et al., 1983). Briefly, soil samples were sieved through a 0.6 mm mesh. A one-gram sample was suspended in 9 mL of sterile water. After incubation at 55°C for 20 min, the soil homogenate was diluted with sterile water into 10^−1^, 10^−2^, and 10^−3^ and poured into the SCA plates. The plates were cultured at 28°C for 7 d, and the colonies with different shapes and colors were picked up and further purified on ISP2 (yeast extract-malt extract agar) (Shirling and Gottlieb, 1966). All the purified isolates were preserved in the ISP2 (yeast malt extract agar) slant agar medium at 4°C and 30% of glycerol (v/v) at −80°C, respectively.

### Screening of antagonistic actinobacteria against Foc TR4

The antifungal activity of actinobacteria was screened against Foc TR4 using the plate confrontation method (Jing et al., 2020). Briefly, these isolates were inoculated at four symmetrical points 2.5 cm away from the center of potato dextrose agar (PDA) plates. After 2 d, the mycelial disk (0.5 cm diameter) of Foc TR4 was inoculated into the center of the PDA plate. The mycelial disk of Foc TR4 only was used as a control. The experiment was set up in triplicate. After incubation at 28°C for 7 d, the diameters of Foc TR4 were measured. The antifungal activity of actinobacteria was calculated according to the following formula: MI = [(C-T)/C] × 100%, where C and T represented the growth diameters of the control and treatment groups, respectively. Strain SCA4-21 (the type strain SCA4-21^T^) was selected to be investigated further due to the strong antifungal activity against Foc TR4.

### Measurement of a broad-spectrum antifungal activity

The broad-spectrum antagonistic activity of strain SCA4-21^T^ was determined using the plate confrontation method as described above. The selected eight pathogenic fungi include *Curvularia fallax* (ATCC 34598), *Colletotrichum gloeosporioides* (Penzig) Penzig et Saccardo (ATCC MYA-456), *Colletotrichum gloeosporioides* (ACCC 36351), *Colletotrichum acutatum* (ATCC 56815), *Colletotrichum fragariae* (ATCC 58718), *Colletotrichum gloeosporioides (Penz) Saec* (ACCC 36351), *Fusarium oxysporum* f. sp. *cucumerinum* Owen (ATCC 204378), and *Fusarium graminearum* (DSM 21803). The pathogens were kept in the Institute of Tropical Bioscience and Biotechnology, China Academy of Tropical Agricultural Sciences, Haikou, China.

### Cultural and morphological characteristics of strain SCA4-21^T^

Strain SCA4-21^T^ was inoculated on eight media including ISP2, ISP3 (oatmeal agar), ISP4 (inorganic salts-starch agar), ISP5 (glycerol-asparagine agar), ISP6 (peptone yeast-iron agar), ISP7 (tyrosine agar), and Gause No. 1 agar and cultured at 28°C for 7 d (Shirling and Gottlieb, 1966). The culture characteristics were observed such as the growth of aerial and substrate hyphae and the production of soluble pigment. The spore chain and spore morphology of strain SCA4-21^T^ were observed by a scanning electron microscope (SEM, Zeiss ∑IGMA, Germany) after 7 and 21 d of growth on ISP2 medium according to our previous method (Qi et al., 2021). Mycelial blocks (5 mm diameter) were cut from plates of strain SCA4-21^T^ using a sterile scalpel. The samples were fixed with 2.5% of glutaraldehyde at 4°C overnight and were rinsed twice with phosphoric acid buffer solution (PBS, 0.1 mol/L, pH 7.0). The materials were dehydrated in a graded ethanol solution (70, 80, 90, 95, and 100%) for 2 min, respectively. The specimens were soaked in fresh t-butyl alcohol overnight. Dehydrated samples were freeze-dried and coated with a film of gold–palladium alloy under vacuum for SEM.

### Physiological and biochemical characteristics of strain SCA4-21^T^

The physiological characteristics of strain SCA4-21^T^ were determined such as the utilization of nitrogen and carbon and the tolerance to NaCl (0–11%, w/v), temperature (16–50°C), and pH (4–10). We also measured its biochemical features, including the production of urease, degradation of Tweens 20, 40, and 60, gelatin liquefaction, starch hydrolysis, nitrate reduction, siderophore, and antibiotic sensitivity test (Qi et al., 2021).

### Chemotaxonomic characteristics of SCA4-21^T^

Strain SCA2-4 ^T^ was inoculated in the ISP2 liquid culture medium and shaken at 180 rpm and 28°C for 5 d. The biomass was collected by centrifugation at 12000 rpm for 5 min. The precipitate was freeze-dried. The cellular fatty acids were determined by gas chromatography (Agilent 6,890) using the Sherlock Microbial Identification System (MIS) software (Sasser, 1990). The composition of cell wall diaminopimelic acid (DAP) was analyzed according to the method of Hasegawa et al. (1983). Respiration quinone was performed as described by Minnikin et al. (1984).

### Genome sequencing and feature analysis of strain SCA4-21^T^

Strain SCA4-21^T^ was cultured in ISP2 liquid medium at 28°C for 3 d. Mycelia of strain SCA4-21^T^ were collected by centrifugation and frozen in nitrogen. The whole genome was sequenced using the Illumina Hiseq + PacBio sequencing platform (Majorbio Bio-Pharm Technology Co., Ltd., Shanghai, China). Raw reads were trimmed and filtered using the Trimmomatic (v 0.36) software (Bolger et al., 2014). The clean reads were assembled using the Unicycler (v 0.4.8) software (Wick et al., 2017). The protein-coding genes were predicted using the Glimmer (v3.02) (Delcher et al., 2007), Prodigal (v 2.6.3) (Hyatt et al., 2010), and GeneMarkS (v4.3) (Besemer and Borodovsky, 2005). Genome annotation was performed by the Prokaryotic Genome Annotation Pipeline of NCBI (Tatusova et al., 2016). CAZymes were first predicted using the online CAZy v 6 software (Cantarel et al., 2009).

### Phylogenetic analysis of strain SCA4-21^T^

Strain SCA4-21^T^ was inoculated in the ISP2 liquid medium and shaken at 180 rpm and 28°C for 3 d. DNA was extracted using the bacterial genome rapid DNA kit (RN43, Aidlab Co., Ltd., Beijing, China). The 16S rRNA sequence was amplified using a pair of the common primers 27F (5’-AGAGTTTGATCCTGGCTCAG-3′) and 1492R (5’-GGTTACCTTGTTACGACTT-3′) (Wang et al., 2015). The PCR reaction system was set according to Wang et al. (2022). After sequencing, the multiple alignment of the 16S rRNA sequence was carried out using EzBioCloud (Hall, 1999)^2^ and CLUSTALW within BioEdit 7.0.5.3 (Thompson et al., 1994). Phylogenetic trees were constructed using neighbor-joining, maximum parsimony, and maximum likelihood methods in MEGA7.0 (Kumar et al., 2016). Multilocus sequence analysis (MLSA) was performed based on five housekeeping genes including *atpD*, *gyrB*, *recA*, *rpoB,* and *trpB* (Rong and Huang, 2010). These housekeeping genes of strain SCA4-21^T^ were extracted from the sequenced genome. The homolog sequences of five housekeeping genes from other species were downloaded from GenBank.^3^ The phylogenetic trees were constructed using the above method. The evolutionary distance was calculated by using the Kimura two-parameter (K2P) model for nucleotide sequences (Kimura, 1980). Based on the alignment of the whole genomes with other type strains of *Streptomyces*, the phylogenomic tree was constructed using the Type (Strain) Genome Server (TyGS) with default parameters (Meier-Kolthoff and Göker, 2019).^4^ The GenBank accession numbers of these housekeeping genes and genomes are listed in Supplementary Table S1.

### The overall genome-related indexes

To further identify the species of strain SCA4-21^T^, the overall genome-related indexes (OGRIs), such as average nucleotide identity (ANI) and digital DNA–DNA hybridization (dDDH), between strain SCA4-21^T^ and its closely related species were calculated by the online OrthoANI (Yoon et al., 2017)^5^ and the Genome-to-Genome Distance Calculator (GGDC) version 3.0 (Meier-Kolthoff et al., 2022),^6^ respectively.

### Determination of antifungal activity of strain SCA4-21^T^ fermentation broth

Antifungal activity of fermentation broth of *Streptomyces* sp. SCA4-21^T^ was determined by the agar well diffusion method (Magaldi et al., 2004). Briefly, strain SCA4-21^T^ was inoculated into 100 mL of liquid YE medium and cultured at 28°C and 180 rpm for 7 d. Culture supernatant was harvested from the culture media of strain SCA4-21^T^ by centrifugation at 2000 rpm for 20 min, followed by filtration through a 0.22-μm sterile filter (Millipore, Bedford, MA, United States). A total of 100 μL of sterile supernatant was aliquoted into each symmetrical well 2.5 cm away from the center of the PDA plate. The mycelial disk (5 mm diameter) of Foc TR4 was placed on the center of the PDA plate. Sterilized H_2_O was used as a control. Three replicates were set for each experiment. After incubating at 28°C for 5–7 d, the antifungal activity of fermentation broth was calculated using the formula MI = [(C-T)/C] × 100%, where C and T expressed the growth diameters of the control and treatment groups, respectively.

### Chitinase and β-1,3-glucanase activity analysis of fermentation broth of strain SCA4-21^T^

The activities of CAZymes, including chitinase and glucanase, were measured. Briefly, a 5-mm block of strain SCA4-21^T^ was inoculated into 100 mL of liquid YE medium and shaken at 28°C and 180 rpm for 7 d. Culture filtrates were collected from the culture media of strain SCA4-21^T^ by centrifugation at 2000 rpm for 20 min. The activities of chitinase and β-1,3-glucanase were determined using the Chitinase Activity Assay Kit (Beijing Solarbio Science & Technology Co., Ltd.) and the microorganism β-1,3-glucanase ELISA Kit (Jiangsu Meibiao Biotechnology Co., Ltd.), respectively. The amount of chitinase that breaks down chitin to produce 1 μg of N-acetylglucosamine per milliliter of sample solution per minute at 37°C is defined as one unit of chitinase activity. One unit of β- 1,3-glucanase activity is expressed as the amount of enzyme that converts 1 μmol of substrate into product per milliliter of sample solution per minute at 37°C. All experiments were repeated three times.

## Results

### Isolation and antifungal activity screening of actinobacteria against Foc TR4

**Figure 1:**
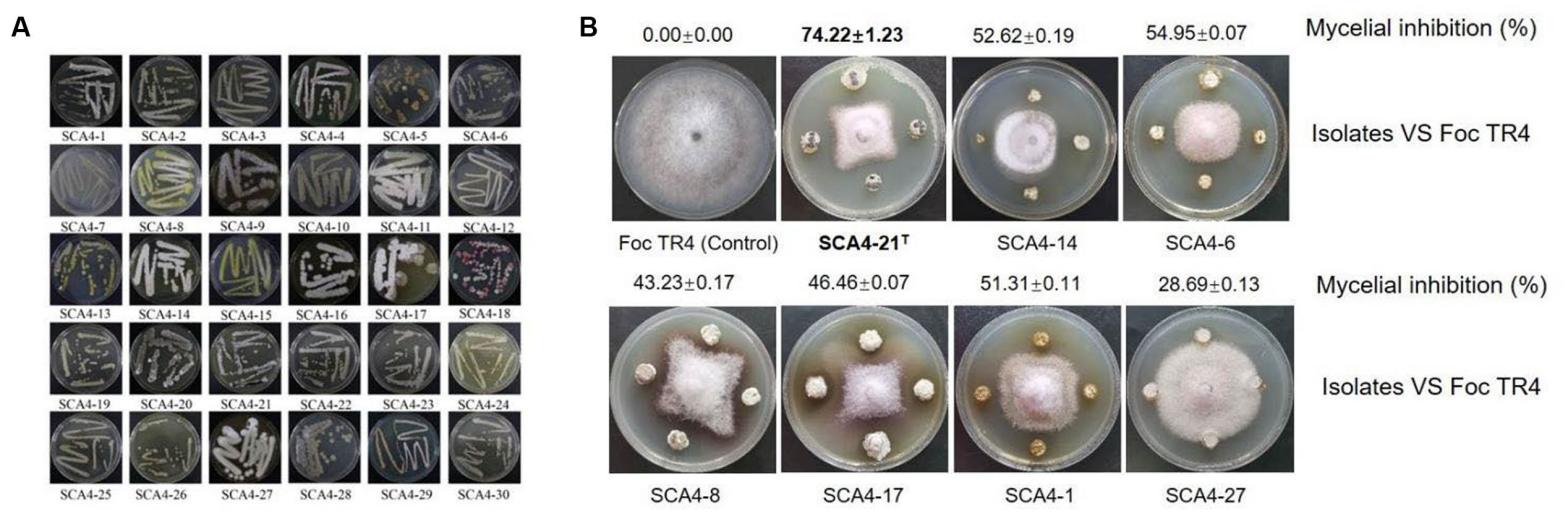
Isolation of actinobacteria and screening of their antagonistic activity against Foc TR4 **(A)** Thirty actinobacteria were isolated from wheat rhizosphere soil samples. **(B)** Seven actinobacteria exhibited antifungal activity against Foc TR4.

A total of 30 actinobacteria were isolated from soil samples in the dry-hot valley and designated as SCA4-1 to SCA4-30 (Figure 1A). The antifungal activities of these isolates against Foc TR4 were analyzed using the plate confrontation method. Seven strains showed antifungal activity against Foc TR4. Among them, SCA4-21^T^ (the type strain SCA4-21^T^) has the highest percentage of mycelial inhibition (74.22 ± 1.23) %, followed by SCA4-6 (54.95 ± 0.07) %, SCA4-14 (52.62 ± 0.19)%, SCA4-1 (51.31 ± 0.11)%, SCA4-17 (46.46 ± 0.07)%, SCA4-8 (43.23 ± 0.17)%, and SCA4-27 (28.69 ± 0.13) %. Therefore, strain SCA4-21^T^ was selected for the following experiment (Figure 1B).

### Strain SCA4-21^T^ exhibited a broad-spectrum antifungal activity

**Figure 2:**
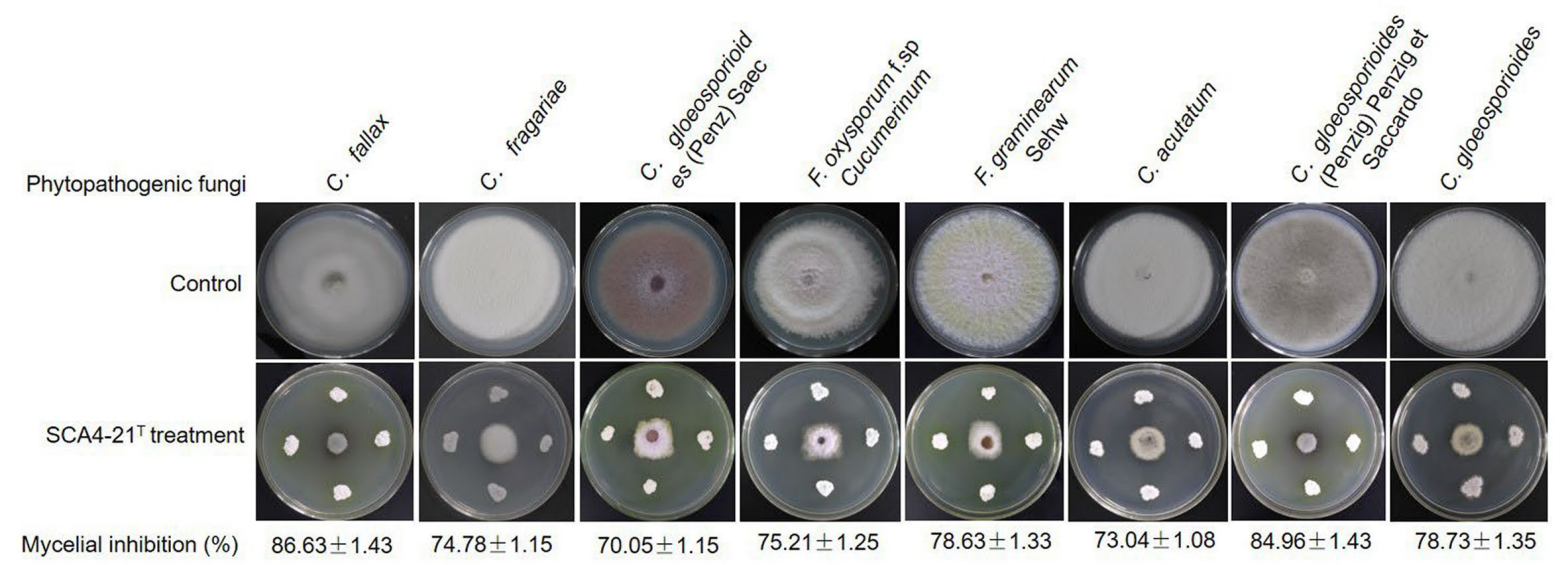
Strain SCA4-21^T^ exhibited broad-spectrum antifungal activity against eight other Phytopathogenic fungi.

Compared with the control group, strain SCA4-21^T^ exhibited a strong broad-spectrum antifungal activity. The highest inhibition rates were observed in *C. fallax* (86.63 ± 1.43)% and *C. gloeosporioides* (Penzig) Penzig et Saccardo (84.96 ± 1.43)%, respectively (Figure 2), followed by *C. gloeosporioides* (78.73 ± 1.35)%, *F. graminearum* Sehw (78.63 ± 1.33)%, *F. oxysporum* f. sp. *cucumerinum* (75.21 ± 1.25)%, *C. fragariae* (74.78 ± 1.15)%, *C. acutatum* (73.04 ± 1.08)%, and *C. gloeosporioides* (Penz) Saec (70.05 ± 1.15)%.

### Cultural and morphological characteristics of strain SCA4-21^T^

**Figure 3:**
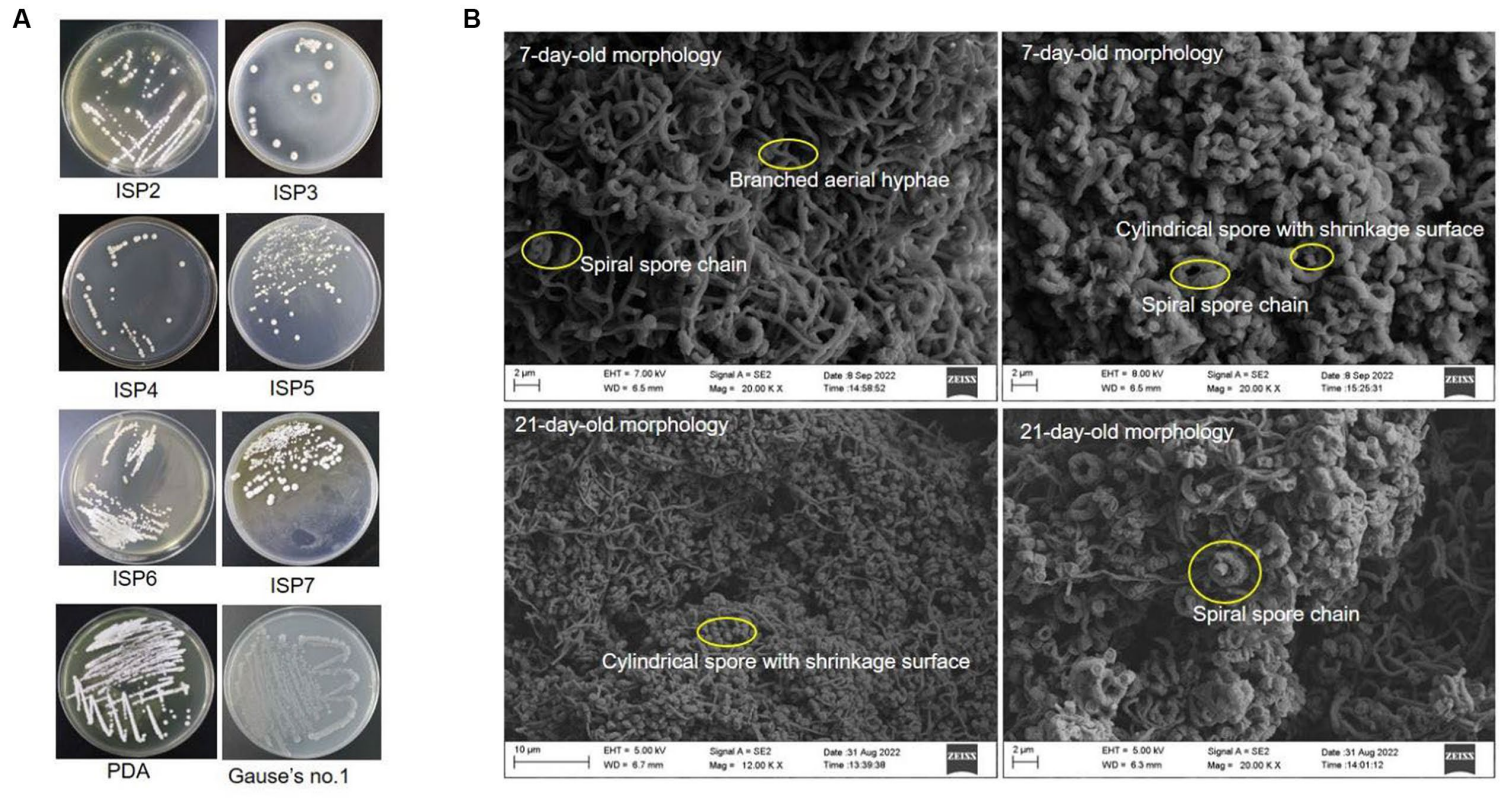
Cultural and morphological characteristics of strain SCA4-21^T^ **(A)** Cultural characteristics of strain SCA4-21^T^ cultured on eight different media, including ISP2, ISP3, ISP4, ISP5, ISP6, ISP7, PDA, and Gause’s no.1 medium. **(B)** Morphological characteristics of strain SCA4-21^T^ cultured on ISP2 plates for 7d and 14 d at 28°C.

Strain SCA4-21^T^ was an aerobic and Gram-positive bacterium. It could grow well on the ISP2, PDA, and Gause’s no.1 plates, followed by ISP5-7. The colors of colonies on different media exhibited white, cream, pure white, gray white, and silver gray. Strain SCA4-21^T^ produced light-brown and light-yellow soluble pigment only in ISP7 and PDA plates, respectively (Figure 3A and Table 1). Morphological observations of strain SCA4-21^T^ on ISP2 plates revealed the presence of branched aerial mycelia, a spiral spore chain, and cylindrical spores with a shrinkage surface. On 7-day-old plates, abundant aerial mycelia and spore chains were observed, while on 21-day-old plates, a large number of mature spores detached from spore chains were observed (Figure 3B). On ISP3, PDA, and Gause’s no.1 plates, these spirals gradually merged into dark masses of spores as they aged. This phenomenon was frequently observed in the members of *Streptomyces hygroscopicus* and *Streptomyces iranensis* groups (Shirling and Gottlieb, 1972; Hamedi et al., 2010). The cultural and morphological characteristics of strain SCA4-21^T^ were consistent with those of the genus *Streptomyces*.

**Table 1:** Cultural characteristics of strain SCA4-21^T^ on eight media.

Culture medium	Aerial hyphae	Vegetative mycelium	Soluble pigment	Colony characteristics	Growth conditions
ISP2	White	Sand yellow	None	Wrinkled, hard	+++
ISP3	Cream	Light ivory	None	Wrinkled, hard	+
ISP4	Pure white	Cream	None	compact, wrinkle-free	+
ISP5	Pure white	Cream	None	compact, wrinkle-free	++
ISP6	Pure white	Cream	None	Loose, wrinkle-free	++
ISP7	Gray white	Sand yellow	Light brown	Loose, wrinkle-free	++
PDA	Pure white	Lemon yellow	Light yellow	Loose, wrinkle-free	+++
Gause’s no.1	Silver gray	Silver gray	None	Loose, powery	+++

“+++” represented that the strain grew well; “+ +” represented the general growth of the strain; “+” represented that the strain could grow.

### Physiological and biochemical characteristics of strain SCA4-21^T^

**Figure 4:**
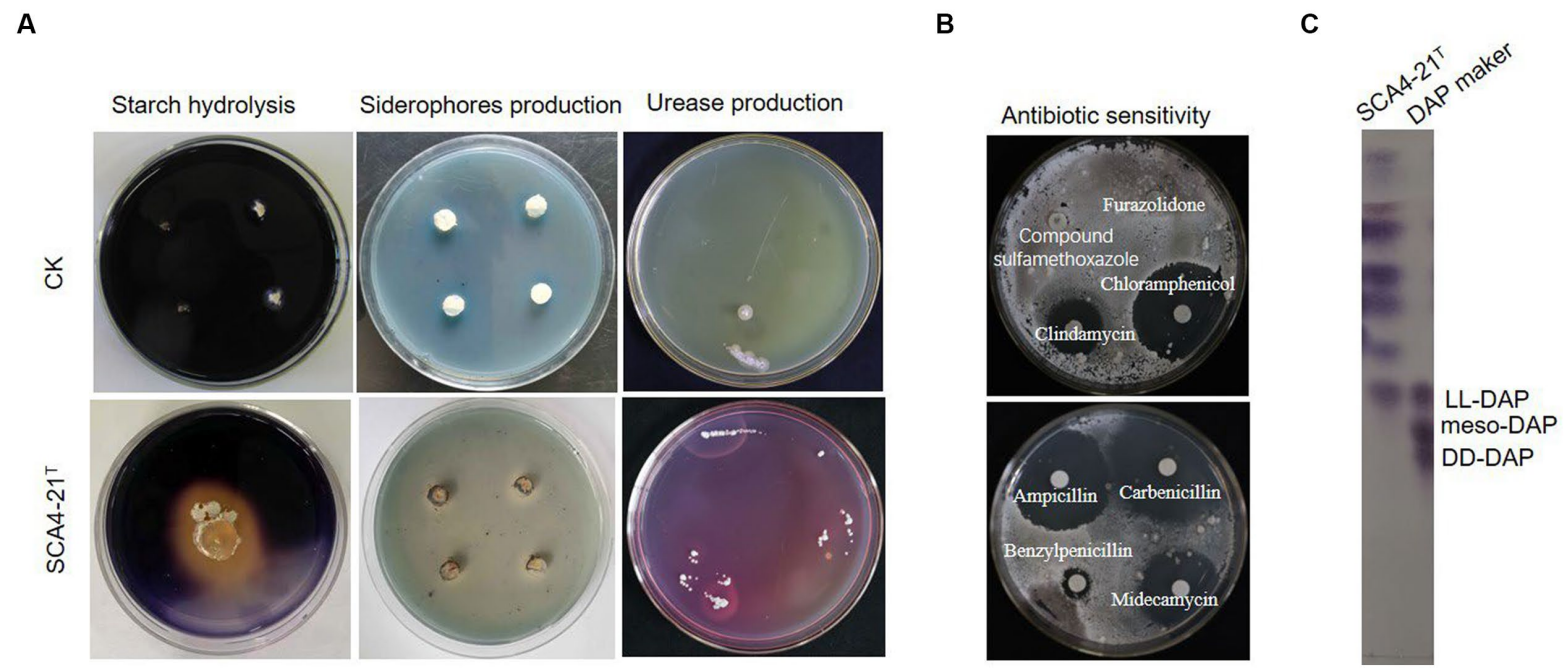
Physiological and chemotaxonomic characteristics of strain SCA4-21^T^ **(A)** Compared with the control strain, strain SCA4-21^T^ could produce amylase, siderophores, and urease. **(B)** Antibiotic sensitivity experiment. The strain was resistant to some antibiotics such as furazolidone, compound sulfamethoxazole, but sensitive to other antibiotics like clindamycin, chloramphenicol, carbenicillin, midecamycin, ampicillin, and benzylpenicillin. **(C)** Composition analysis of DAP in cell wall. The cell wall of strain SCA4-21^T^ mainly contained LL-DAP.

The growth of strain SCA4-21^T^ was observed at pH 6.0 to 9.0 (optimal at 7), at temperature 16–46°C(optimal at 28°C), and with NaCl of 0 to 3% (w/v) (optimal at 2%). It could degrade starch, urea, and Tween-20 while producing melanoid pigment and siderophores (Figure 4A). It could neither degrade gelatin, Tween 40, Tween 60, nitrate, and cellulose nor produce H_2_S (Supplementary Table S2). The strain demonstrated resistance to 10 antibiotics, namely furazolidone, compound sulfamethoxazole, polymyxin B, vancomycin, erythromycin, minocycline, kanamycin, gentamicin, ceftriaxone, and cefuroxime. However, it was sensitive to 11 antibiotics, namely, clindamycin, chloramphenicol, penicillin, cefepime, cefotaxime, cefamandole, midecamycin, carbenicillin, ampicillin, benzylpenicillin, and piperacillin (Figure 4B; Supplementary Table S2). In addition, as shown in Supplementary Table S3, the isolate was able to utilize D-mannose, D-trehalose, sorbitol, D-fructose, lactose, mannitol, and xylan as carbon sources but could not use L-arabinose and raffinose. Strain SCA4-21^T^ used L-phenylalanine, L-asparagine, L-methionine, L-valine, L-histidine, L-tryptophan, D-cellobiose, L-hydroxyproline, and L- cysteine but did not utilize anhydrous inositol, L-glutamic acid, L-tyrosine, and arginine.

### Chemotaxonomic characteristics of strain SCA4-21^T^

The chemotaxonomic characteristics of strain SCA4-21^T^ are listed in Table 2. The dominant menaquinones were MK9 (H8) (65.50%) and MK10(H2) (34.50%) (Supplementary Figure S1). The dominant fatty-acid compositions of the isolate consisted of anteiso-C_15:0_ (34.78%) and C_16:0_ (19.89%). The presence of LL-DAP in the peptidoglycan of the cell wall was considered one of the effective methods for the identification of the genus *Streptomyces* (Kieser et al., 2000). The cell wall of strain SCA4-21^T^ largely contained LL-diaminopimelic acid (LL-DAP) (Figure 4C), which helped in the rapid identification of this strain as *Streptomyces*.

**Table 2:** Chemotaxonomic characteristics of strain SCA4-21^T^.

Characteristic	SCA4-21
Major menaquinones (%)	
MK9(H_8_)	**65.50**
MK10(H_2_)	**34.50**
Major fatty acids (>0.5%)	
iso-C_13:0_	0.83
anteiso-C_13:0_	0.59
iso-C_14:0_	8.21
C_14:0_	3.34
iso-C_15:0_	9.67
anteiso-C_15:0_	**34.78**
C_16:0_	**19.89**
iso-C_16:1_ H	tr
iso-C_16:0_	8.16
anteiso-C_16:0_	ND
iso-C_17:0_	1.4
anteiso-C_17:0_ *ω9c*	0.53
anteiso-C_17:0_	5.93
C_17:0_*cyclo*	1.78
C_17:0_	0.75
iso-C_18:0_	tr
Summed feature 3*	0.54
Summed feature 9*	tr
The main amino acid of the cell wall	LL-diaminopimelic acid

Percentages of the total fatty acids and fatty acids amounting to less than 0.5% in all strains were not shown. Major components (> 10.0%) were highlighted in bold. tr, trace amount (< 0.5%); ND, not detected. *Summed features represented the groups of two fatty acids that cannot be separated by gas–liquid chromatography with the MIDI system. Summed feature 3* comprised C16:1 ω6c and/or C16:1 ω7c, Summed feature9* comprised iso-C17:1 ω9c and/or C16:0 10-methyl.

### Genome sequencing and feature analysis of strain SCA4-21^T^

**Figure 5:**
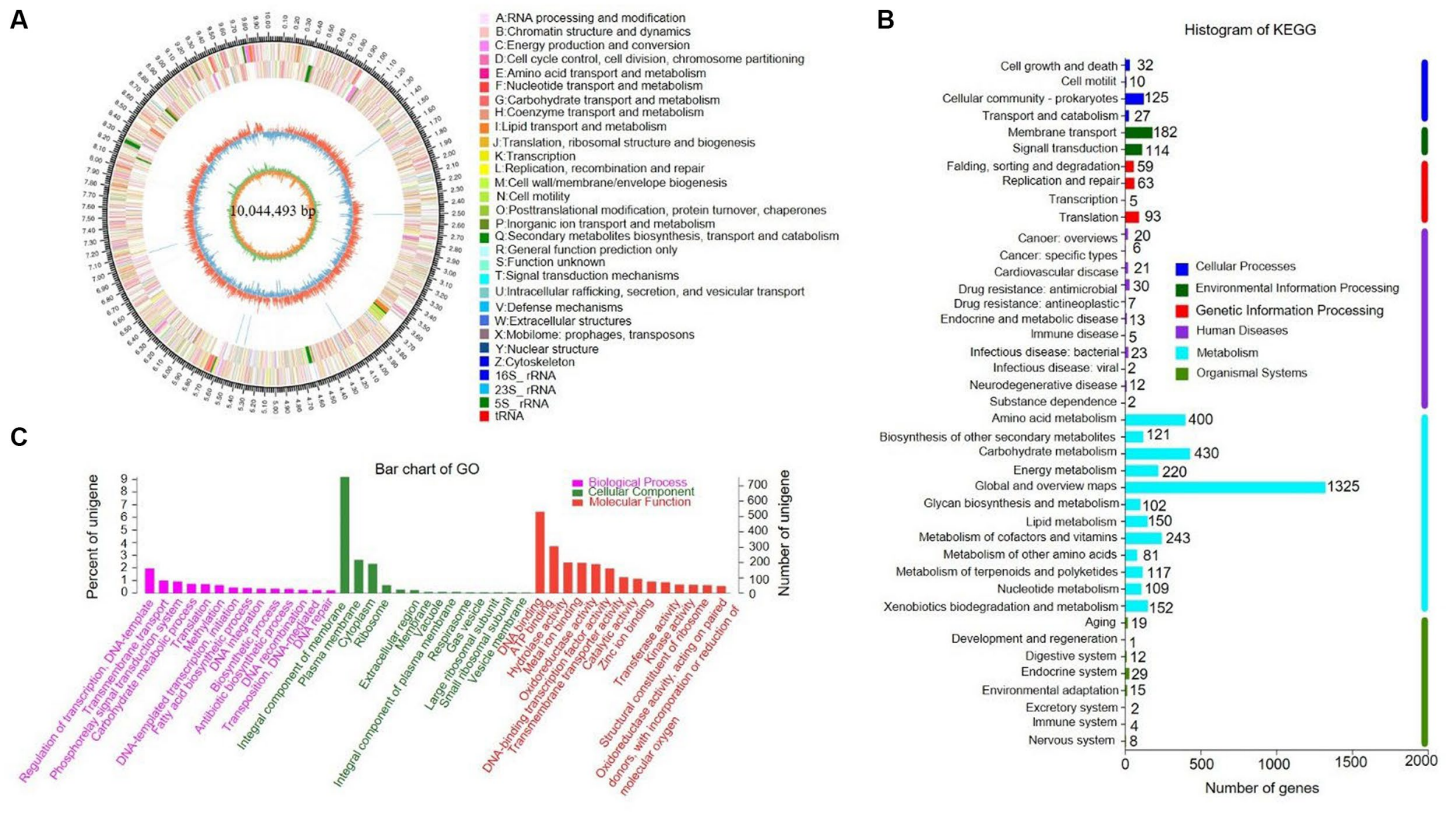
Genome information and function annotation of strain SCA4-21^T^ **(A)** Circular map. From the outside to the middle, ring 1 was the mark of genome size. Rings 2 and 3 represent CDS on forward/reverse strand. Different colors indicate the functional categories of CDS. Ring 4 is tRNA and rRNA. Ring 5 is the G + C content. The outward red portion indicates that the GC content of this region is higher than the average GC content of the whole genome. The inward blue part indicates that the GC content of this region is lower than the average GC content of the whole genome, followed by G + C skew in ring 6. **(B)** The KEGG pathway annotation. **(C)** The GO annotation.

The complete genome of strain SCA4-21^T^ was sequenced and assembled into a circular chromosome of 10,044,493 bp with 71.48% of GC content (Figure 5A). Genomic features of strain SCA4-21^T^ are listed in Table 3. The genome contained 8,246 protein-coding genes (CDS) with 8,847,075 bp of gene total length, 64 tRNA genes, 18 rRNA genes, 134 sRNA genes, 1,436 repeat genes, 80 CRISPR-Cas genes, and 6 insert sequences. Functional analysis revealed that 7,548, 3,460, and 4,391 genes were annotated to KEGG, COG, and GO categories, respectively (Figures 5B,C). In COG function classification, most of the predicted CDS related to metabolism (47.4%), followed by cellular processes and signaling (20.2%) and information storage and processing (19.7%). A total of 12.6% of CDS are poorly characterized (Supplementary Table S4).

**Table 3:** Genome features of strain SCA4-21^T^.

Feature	Chromosome characteristics
Genome size (bp)	10,044,493
Chromosome No.	1
Plasmid No.	0
GC Content (%)	71.48
Depth	454.86
Protein-coding genes No.	8,246
Gene total length (bp)	8,847,075
Gene average length (bp)	1072.89
Gene density	0.82
tRNA genes No.	64
Type of tRNAs No.	20
rRNA genes No.	18
16S rRNA No.	6
23S rRNA No.	6
5S rRNA No.	6
sRNA No.	134
Repeat No.	1,436
CRISPR-Cas No.	80
Insert sequences No.	6
Genes assigned to COG (bp)	7,548
Genes assigned to KEGG (bp)	4,930
Genes assigned to GO (bp)	3,460

### Phylogenetic analysis of strain SCA4-21^T^

**Figure 6:**
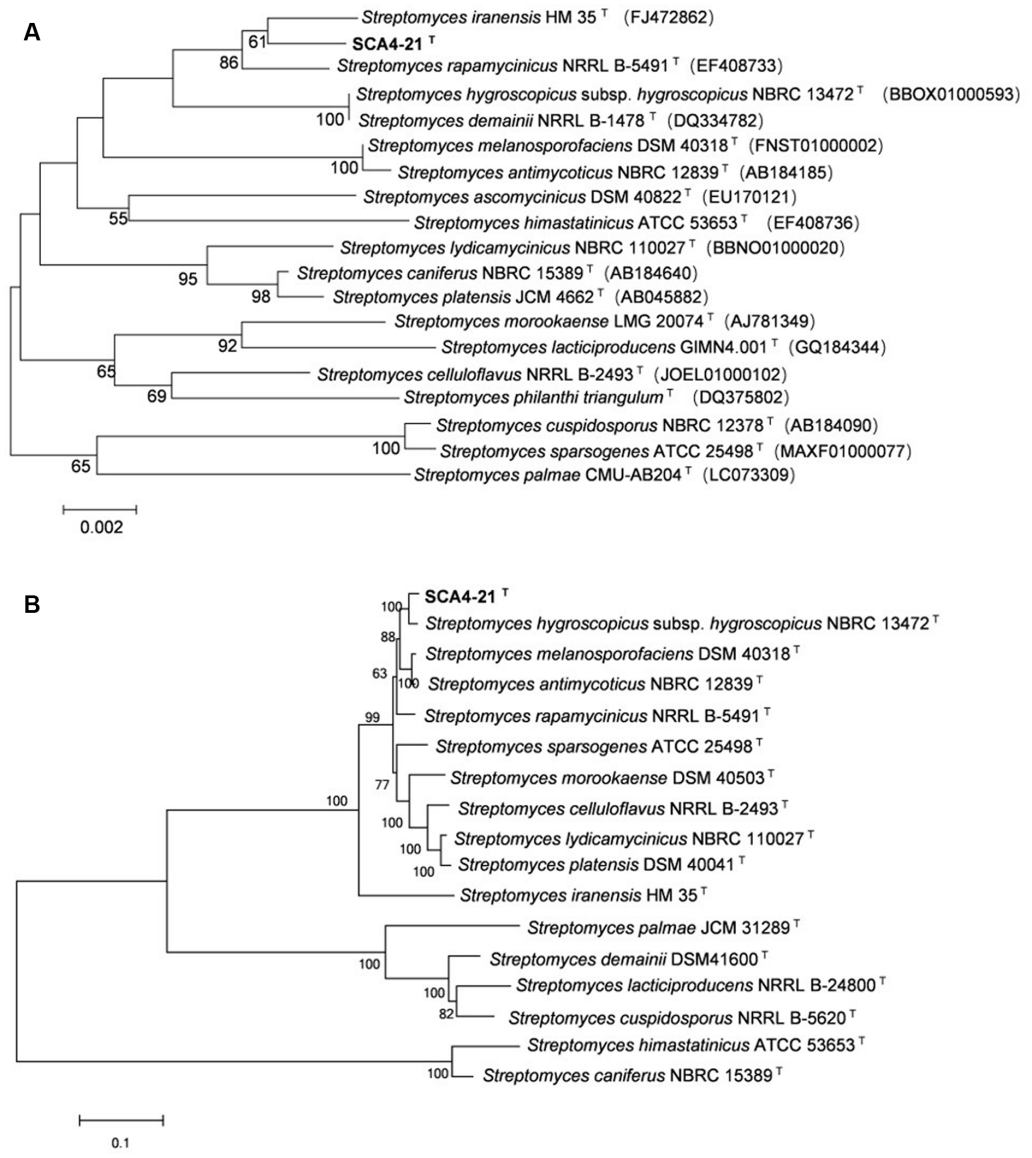
The phylogenetic trees of strain SCA4-21^T^ **(A)** The phylogenetic tree based on the complete 16S rRNA sequences (1,524 bp) was constructed using neighbor-joining method. Accession numbers of the selected genes were listed in brackets. **(B)** The phylogenetic tree based on concatenated five housekeeping genes (*atpD*, *gyrB*, *recA*, *rpoB*, and *trpB*) (9,381 bp) was constructed using neighbor-joining method. Strain names and accession numbers are listed in Supplementary Table S1. Bootstrap percentages (based on 1,000 replications) were shown at branching points. Bar, 0.002 or 0.1 substitutions per nucleotide position.

The EzBioCloud analysis of the 16S rRNA gene sequence (1,524 bp) of strain SCA4-21^T^ revealed that the strain shared the highest similarity to *S. iranensis* HM 35^T^ (99.45%), *S. rapamycinicus* NRRL B-5491^T^ (99.35%), and *S. hygroscopicus* subsp*. hygroscopicus* NBRC 13472^T^ (99.17%). The phylogenetic trees based on the 16S rRNA sequences were constructed using neighbor-joining (NJ), maximum parsimony (MP), and maximum likelihood (ML) methods and are shown in Figure 6A; Supplementary Figures S2, S3, respectively. NJ and MP trees revealed that strain SCA4-21^T^ formed a subclade with *S. iranensis* HM 35^T^ with low bootstrap values, indicating that the strain belongs to the genus *Streptomyces* (Figure 6A; Supplementary Figure S2). However, the ML tree showed that strain SCA4-21^T^ formed a subclade with *S. iranensis* HM 35^T^ and *S. rapamycinicus* NRRL B-5491^T^ with a bootstrap value of 63% (Supplementary Figure S3). The above results indicate that it is difficult to distinguish this strain from its closely related species using the 16S rRNA gene.

Therefore, to assign strain SCA4-21^T^ to species, MLSA was performed using five housekeeping genes: *atpD*, *gyrB*, *recA*, *rpoB*, and *trpB*. The phylogenetic trees using all three algorithms showed that SCA4-21^T^ formed a well-delineated subclade with *Streptomyces hygroscopicae* subsp. *hygroscopicus* NBRC 1372^T^ with bootstrap values of 100% (Figure 6B; Supplementary Figures S4, S5). Therefore, five housekeeping genes, namely, *atpD*, *gyrB*, *recA*, *rpoB*, and *trpB,* could distinguish closely related *Streptomyces* species. The MLSA distance values ranged from 0.021 to 1.092 (Table 4), which is much higher than the threshold of 0.007 for the delineation of bacterial species (Rong and Huang, 2012). The results were also approved by the analysis of the genome-based phylogeny (Supplementary Figure S6). The above data indicated that strain SCA4-21^T^ represents a novel species of *Streptomyces*.

**Table 4:** MLSA distance values for the selected strains.

	MLSA distance (Kimura two-parameter)																
Strains	1	2	3	4	5	6	7	8	9	10	11	12	13	14	15	16	17
1																	
2	0.187																
3	0.050	0.153															
4	0.021	0.188	0.048														
5	0.679	0.714	0.705	0.660													
6	0.040	0.180	0.041	0.040	0.676												
7	0.039	0.178	0.041	0.040	0.677	0.008											
8	1.092	1.136	1.100	1.077	1.102	1.104	1.094										
9	0.092	0.226	0.093	0.092	0.710	0.089	0.088	1.099									
10	1.038	1.091	1.039	1.025	1.080	1.029	1.027	0.105	1.040								
11	0.098	0.232	0.098	0.096	0.715	0.093	0.093	1.102	0.018	1.050							
12	0.094	0.231	0.094	0.094	0.728	0.086	0.089	1.120	0.047	1.057	0.053						
13	0.093	0.221	0.089	0.091	0.711	0.088	0.087	1.120	0.087	1.041	0.093	0.089					
14	0.754	0.735	0.729	0.738	0.119	0.717	0.718	1.186	0.734	1.130	0.736	0.739	0.713				
15	0.731	0.744	0.734	0.735	0.276	0.725	0.727	1.234	0.745	1.178	0.747	0.761	0.735	0.297			
16	0.714	0.726	0.708	0.707	0.084	0.696	0.694	1.133	0.723	1.091	0.732	0.747	0.724	0.109	0.298		
17	0.065	0.204	0.068	0.063	0.685	0.067	0.068	1.085	0.094	1.031	0.100	0.103	0.092	0.732	0.729	0.672	

Strains: 1. SCA4-21^T^, 2. *Streptomyces iranensis* HM 35^T^, 3. *Streptomyces rapamycinicus* NRRL B-5491^T^, 4. *Streptomyces hygroscopicus* subsp. *hygroscopicus* NBRC 13472^T^, 5. *Streptomyces demainii* DSM41600^T^, 6. *Streptomyces melanosporofaciens* DSM 40318^T^, 7. *Streptomyces antimycoticus* NBRC 12839^T^, 8. *Streptomyces himastatinicus* ATCC 53653^T^, 9. *Streptomyces lydicamycinicus* NBRC 110027^T^, 10. *Streptomyces caniferus* NBRC 15389^T^, 11. *Streptomyces platensis* DSM 40041^T^, 12. *Streptomyces celluloflavu* NRRL B-2493^T^, 13. *Streptomyces morookaense* DSM 40503^T^, 14. *Streptomyces lacticiproducens* NRRL B-24800^T^, 15. *Streptomyces palmae* JCM 31289^T^, 16. *Streptomyces cuspidosporus* NRRL B-5620^T^, 17. *Streptomyces sparsogenes* ATCC2549^T^.

### The overall genome-related indexes

The ANI and dDDH values were calculated between strain SCA4-21^T^ and closely related species. The species were selected based on the results of the phylogenetic trees constructed using the 16S rRNA gene and five housekeeping genes. Strain SCA4-21^T^ showed the highest ANI and dDDH values with *Streptomyces hygroscopicus* subsp. *hygroscopicus* NBRC 13472^T^, which were 91.26 and 48%, respectively (Table 5). These values were below the novel species threshold of 95% (ANI) and 70% (dDDH) (Meier-Kolthoff et al., 2022; Riesco and Trujillo, 2024). These findings further supported that strain SCA4-21^T^ belongs to a novel species in the genus *Streptomyces*.

**Table 5:** The ANI and dDDH values between strain SCA4-21^T^ and its closely related species.

Strains	ANI (%)	dDDH (%)
*Streptomyces iranensis* DSM 41954^T^	89.42	37.8
*Streptomyces rapamycinicus* NRRL B-5491^T^	89.54	38.1
*Streptomyces hygroscopicus* subsp. *hygroscopicus* NBRC 13472^T^	91.26	44.3
*Streptomyces melanosporofaciens* DSM 40318^T^	89.95	39.2
*Streptomyces antimycoticus* NBRC 12839^T^	89.8	39
*Streptomyces demainii* DSM 41600^T^	89.25	43.5
*Streptomyces himastatinicus* ATCC 53653^T^	84.73	28.5

### Cazymes prediction of the genome of strain SCA4-21^T^

**Figure 7:**
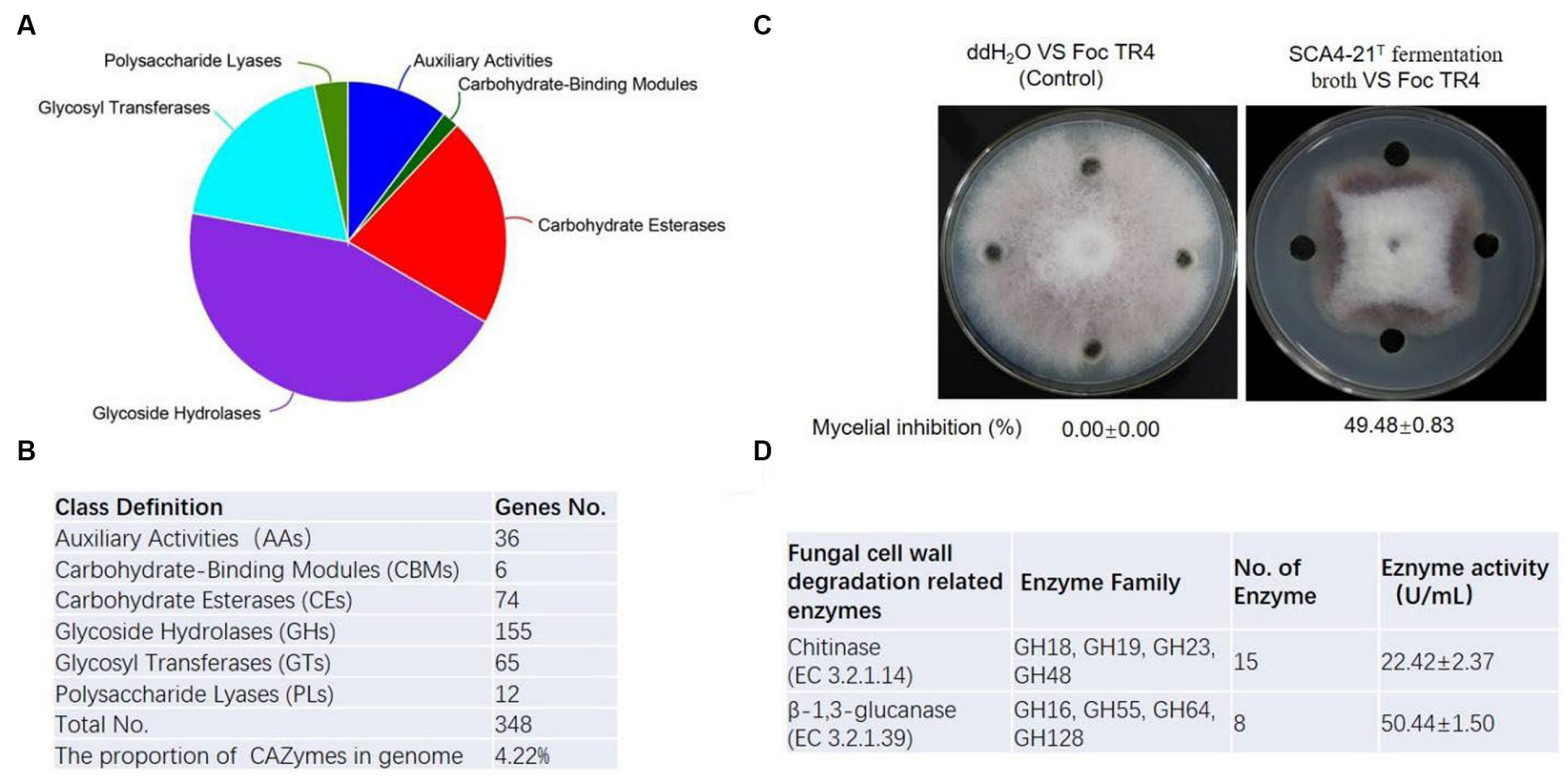
CAZymes analysis of strain SCA4-21^T^ **(A)** Pie chart of CAZymes **(B)** The number of genes in different families of CAZymes. **(C)** Determination of antifungal activity in fermentation broth of SCA4-21^T^. **(D)** Evaluation of chitinase and β-1,3-glucanase activity in fermentation broth of SCA4-21^T^.

To predict enzyme genes related to fungal cell wall degradation, CAZymes of strain SCA4-21^T^ were annotated (Figure 7A). The results demonstrated that the genome of strain SCA4-21^T^ encoded 348 CAZymes including 36 auxiliary activities (AAs), 6 carbohydrate-binding modules (CBMs), 74 carbohydrate esterases (CEs), 155 glycoside hydrolases (GHs), 65 glycosyltransferases (GTs), and 12 polysaccharide lyases (PLs). The proportion of CAZymes was 4.22% in strain SCA4-21^T^ genome (Figure 7B). Family and gene ID of strain SCA4-21^T^ CAZymes are listed in Supplementary Table S5. AAs were divided into nine families, namely AA1, AA2, AA3, AA3_2, AA4, AA5, AA6, AA7, and AA10. CBMs contained four families: CBM2, CBM13, CBM35, and CBM66. CEs included 11 families: CE1, CE2, EC3, EC4, EC7, EC8, EC9, EC10, EC12, EC14, and EC15. GTs were classified into 15 families: GT1, GT2_Glycos_transf_2, GT2_Glyco_tranf_2_3, GT4, GT5, GT9, GT20, GT28, GT35, GT39, GT41, GT76, GT81, GT83, and GT87. PLs were divided into 11 families: PL1, PL1_5, PL1_6, PL3_4, PL7_3, PL8, PL9, PL9_3, PL11, PL26, and PL31. GHs were classified into 80 families: GH1, GH2, GH3, GH4, GH5_1, GH5_8, GH5_18, GH5_19, GH5_40, GH5_43, GH6, GH8, GH9, GH10, GH11, GH12, GH13_3, GH13_9, GH13_10, GH13_11, GH13_13, GH13_16, GH13_26, GH13_30, GH13_32, GH15, GH16, GH18, GH19, GH20, GH23, GH25, GH26, GH27, GH30_5, GH30_7, GH31, GH33, GH35, GH36, GH42, GH43, GH43_3, GH43_5, 1 GH43_10, GH43_24, GH43_26, GH43_34, GH44, GH46, GH48, GH51, GH54, GH55, GH62, GH63, GH64, GH65, GH67, GH74, GH76, GH77, GH78, GH85, GH87, GH89, GH92, GH93, GH95, GH106, GH109, GH113, GH114, GH127, GH128, GH135, GH145, GH146, GH152, and GH154. Fifteen Chitinase (EC 3.2.1.14) were identified in GH18, GH19, GH23, and GH48 families. Additionally, eight β-1,3-glucanases (EC 3.2.1.39) were found in GH16, GH55, GH64, and GH128 families (Figure 6D). Chitinase and β-1,3-glucanases are often found to degrade the cell walls of plant pathogenic fungi (Fan et al., 2002; Arora et al., 2007). Therefore, we suggest that chitinase and β-1,3-glucanases could be responsible for the antifungal activity of strain SCA4-21^T^.

### Antifungal activity analysis of fermentation broth of strain SCA4-21^T^

To further analyze the antifungal activity of the fermentation broth from strain SCA4-21^T^, the inhibition percentage against Foc TR4 mycelia was measured. By determination, the inhibition rate was 49.48 ± 0.83% (Figure 7C). The activities of chitinase and β-1,3-glucanase were 22.42 ± 2.37 U/mL and 50.44 ± 1.50 U/mL in the fermentation broth, respectively (Figure 6D), suggesting that two enzymes could be contributed to the antifungal activity against Foc TR4.

### Description of *Streptomyces luomodiensis* sp. nov

*Streptomyces luomodiensis* (luo.mo.di.en’sis. N.L. masc./fem. Adj. luomodiensis of Luomodi, a village in China, referring to the place where the type strain was first isolated).

Gram-positive, aerobic, and non-motile actinobacteria forming branched substrate and aerial mycelia, which differentiate into spiral spore chains. Spores are cylindrical with a shrinkage surface. Abundant growth occurs on all ISP, PDA, and Gause’s no.1 plates, and the colonies are white, pure or gray white, silver gray, and cream. Growth appears at 20–46°C with an optimum of 28°C, at pH 6–9 with an optimum of 7.0, and 0–3% (w/v) NaCl concentration with an optimum of 2%. Soluble pigment forms on ISP7 and PDA media. They degrade urea, Tween 20, and starch while producing melanoid pigment and siderophores. They neither degrade gelatin, Tween 40 and 60, nitrate, or cellulose nor produce H_2_S. D-mannose, D-trehalose, sorbitol, D-fructose, lactose, mannitol, and xylan are utilized as sole source of carbon and L-phenylalanine, L-asparagine, L-methionine, L-valine, L-histidine, L-tryptophan, D-cellobiose, L-hydroxyproline, and L- cysteine are utilized as sole source of nitrogen. The predominant menaquinones are MK9 (H8) and MK10 (H2). Major fatty acids (>10.0%) are anteiso-C15:0 and C16:0. Cell wall hydrolysates contain LL-diaminopimelic acid (LL-DAP), but they do not contain meso-DAP or DD-DAP.

The type strain, SCA4-21^T^ (=GDMCC4.340^T^ = JCM36555^T^), was isolated from the soil of Luomodi Village, Huili County, Sichuan Province, China. The genome of the type strain is characterized by a size of 10,044,493 bp and a G + C content of 71.48 mol%. Raw reads of strain SCA4-21^T^ were deposited to the SRA database with the accession number SRP303585, and 16S rRNA gene and complete genome sequence were submitted to GenBank with accession numbers OQ352835 and CP117522, respectively.

## Discussion

Banana *Fusarium* wilt caused by Foc TR4 is a destructive fungal disease. More than 80% of bananas are susceptible to Foc TR4 (Pérez-Vicente et al., 2014). The utilization of antagonistic microorganisms for controlling banana *fusarium* wilt has received increasing attention due to its safety and efficacy. To enhance the effectiveness of biological control, it is crucial to screen broad-spectrum and novel antagonistic microorganisms as biocontrol agents (Heydan and Pessarakh, 2010). Unique environments often harbor an abundance of novel microorganisms (Bull, 2010). Here, 30 actinobacteria displaying diverse morphologies were isolated from the dry-hot valley of Huili County (Figure 1A). Among them, seven actinobacteria exhibited antagonistic activity against Foc TR4 (Figure 1B). Especially, strain SCA4-21^T^ showed the most antifungal activity against Foc TR4 and other eight phytopathogenic fungi (Figure 2B).

Fungal cell walls are mainly composed of polysaccharides, accounting for 80–90% of their dry weight. CAZymes are the most important enzymes for polysaccharide degradation. The functional genes encoding CAZymes participate in fungal cell wall degradation. CAZymes analysis demonstrates that the genome of strain SCA4-21^T^ contains 348 CAZymes, accounting for 4.22% of total genes (Figures 7A,B). In most bacterial genomes, CAZymes typically comprise less than 2% of total genes (Mann et al., 2013). The genome of strain SCA4-21^T^ exhibited a high percentage of CAZymes, suggesting its strong potential for degrading fungal cell walls. Specifically, the GH family of CAZymes in strain SCA4-21^T^ genome contained 15 chitinase genes and eight β-1,3-glucanase genes (Figure 7D). These enzymes are responsible for catalyzing the hydrolysis of chitin and β-1,3-glucan, which are the major structural components of fungal cell walls. Accumulated data indicate that chitinases and β-1,3-glucanases produced by biocontrol microbes and plants can inhibit fungal growth (Li et al., 2021; Ruangwong et al., 2022). The study conducted by Arora et al. (2007) showed that the chitinase and β-1,3-glucanase produced by fluorescent pseudomonads can inhibit the growth of *Rhizoctonia solani*. Aktuganov et al. (2008) demonstrated that two enzymes chitinase and β-1,3-glucanase purified from *Paenibacillus ehimensis* IB-X-b have wide-range antifungal activity by degrading fungal mycelia cell walls. Anitha and Rebeeth (2009) reported that chitinase extracted from *Streptomyces griseus* can inhibit soil-borne plant pathogens such as *Fusarium oxysporum*, *Alternaria alternata*, *Rhizoctonia solani*, and *Fusarium solani*. Chitinase and β-1,3-glucanase produced by *Streptomyces cavourensis* SY224 contribute to the biocontrol of *anthracnose* in pepper (Lee et al., 2012). In the present study, the fermentation broth of strain SCA4-21^T^ has high levels of chitinase and β-1,3-glucanase activities (Figure 7C). These findings suggest that these two enzymes could play a crucial role in the antifungal activity of strain SCA4-21^T^ against Foc TR4.

A complete database of 16S rRNA sequences is available for the type strains of prokaryotic species, allowing rapid identification of strains at the genus level. Nonetheless, the phylogeny of the 16S rRNA gene is difficult to distinguish closely related species because of the high level of sequence conservation (Gevers et al., 2005; Guo et al., 2008; Richter and Rosselló-Móra, 2009). The gene clusters of *atpD*, *gyrB*, *recA*, *rpoB*, and *trpB* have a higher level of sequence divergence than that of 16S rRNA genes. MLSA based on these five housekeeping genes has been used as an alternative phylogenetic marker in species discrimination of *Streptomyces* (Rong et al., 2009; Huang et al., 2019; Qi et al., 2021, 2022). In our present study, strain SCA4-21^T^ exhibited the highest similarity to *S. iranensis* HM 35^T^ (99.45%), *S. rapamycinicus* NRRL B-5491^T^ (99.35%), and *S. hygroscopicus* subsp*. hygroscopicus* NBRC 13472^T^ (99.17%). However, the 16S rRNA phylogenetic analysis revealed that strain SCA4-21^T^ clustered with *S. iranensis* HM 35^T^ in the NJ and MP trees, with bootstrap values of 61 and 36%, respectively (Figure 6A; Supplementary Figure S2), while it clustered with *S. iranensis* HM 35^T^ and *S. rapamycinicus* NRRL B-5491^T^ in the ML tree (Supplementary Figure S3). Consequently, the phylogeny of the 16S rRNA gene was unable to effectively differentiate strain SCA4-21^T^ from its closely related species. Therefore, MLSA of this strain based on five housekeeping genes (*atpD, gyrB, recA, rpoB, and trpB*) was performed. The NJ, MP, and ML phylogenetic trees all consistently indicated that strain SCA4-21^T^ had the highest bootstrap values and formed a well-delineated subclade with *S. hygroscopicus* subsp. *hygroscopicus* NBRC 13472^T^ (Figure 6B; Supplementary Figures S4, S5). The phylogenomic tree of strain SCA4-21^T^ by comparing it to the type strains of *Streptomyces* further supported the above findings (Supplementary Figure S6). Therefore, strain SCA4-21^T^ is closely related to *S. hygroscopicus* subsp. *hygroscopicus* NBRC 13472^T^.

After the identification of most closest species, the similarity alignment of the genome is required to assign the isolate to a known or unknown species (Gevers et al., 2005). When it comes to identifying prokaryotic species, the traditional DNA–DNA hybridization (DDH) is time-consuming and labor-intensive, with low inter-laboratory reproducibility (Richter and Rosselló-Móra, 2009). With the rapid development of sequencing technology, it has become possible to directly use the alignment of genome sequences to classify the species. The overall genome-related indexes (OGRI), such as ANI and dDDH, have been widely used to replace the traditional DDH to identify different species (Richter and Rosselló-Móra, 2009; Zhang et al., 2019; Meier-Kolthoff et al., 2022). The ANI and dDDH values for accepted species boundary are around 95–96 and 70%, respectively (Riesco and Trujillo, 2024). In this study, ANI and dDDH values were calculated between strain SCA4-21^T^ and the adjacent seven strains in the phylogenetic trees of the 16S rRNA gene or MLSA. The genome of strain SCA4-21^T^ showed 91.26% of ANI and 44.3% of dDDH with *S. hygroscopicus* subsp. *hygroscopicus* NBRC 13472^T^ (Table 5). Both values are below the respective threshold values, indicating that strain SCA4-21^T^ represents a novel species of *Streptomyces*. These findings offer a novel potential biocontrol agent for the management of plant fungal diseases.

## Conclusion

A total of 30 actinobacteria were isolated and screened for their antagonistic activity against Foc TR4. Strain SCA4-21^T^ exhibited a strong antifungal activity. Based on the morphological, physiological, biochemical, and chemotaxonomic characteristics, strain SCA4-21^T^ was identified as genus *Streptomyces*. The genome of strain SCA4-21^T^ was subjected to complete genome sequencing and assembly, resulting in a 10,044,493 base pair chromosome. This chromosome encodes 8,246 protein-coding genes, with a GC content of 71.48%. CAZymes analysis revealed the presence of 348 carbohydrate-active enzymes, which accounted for 4.22% of the total genes. These included 15 chitinases and 8 β-1, 3-glucanases related to fungal cell wall degradation. The phylogenetic trees of 16S rRNA do not distinguish the close species *Streptomyces.* MLSA showed that strain SCA4-21^T^ clustered with *S. hygroscopicus* subsp. *hygroscopicus* NBRC 13472^T^ with 100% of bootstrap value. The MLSA distance between strain SCA4-21^T^ and *S. hygroscopicus* subsp. *hygroscopicus* NBRC 13472^T^ is 0.021, significantly exceeding the threshold of 0.007 for the delineation of bacterial species, thereby suggesting that strain SCA4-21^T^ represents a novel species. The taxonomic status of strain SCA4-21^T^ was also supported by the phylogenomic tree. ANI and dDDH values are calculated to further verify the conclusion. The strain is named after *Streptomyces luomodiensis* sp. nov. The type strain is SCA4-21^T^ (=GDMCC4.340^T^ = JCM 34963^T^).

## Data availability statement

The datasets presented in this study can be found in online repositories. The names of the repository/repositories and accession number(s) can be found in the article/Supplementary material.

## Author contributions

DQ: Conceptualization, Methodology, Writing – review & editing. QL: Formal analysis, Investigation, Writing – original draft. LiZ: Methodology, Writing – original draft. MZ: Methodology, Writing – original draft. KL: Writing – original draft, Formal analysis. YZ: Writing – original draft, Formal analysis. YC: Visualization, Writing – original draft. JF: Visualization, Writing – original draft. DZ: Writing – original draft, Resources. YW: Writing – original draft, Formal analysis. WW: Funding acquisition, Project administration, Writing – review & editing. LuZ: Writing – review & editing, Resources. JX: Funding acquisition, Project administration, Writing – review & editing.

## Footnotes

^1^ https://www.bacterio.net/

^2^ https://www.ezbiocloud.net/

^3^ https://www.ncbi.nlm.nih.gov/

^4^ https://tygs.dsmz.de/

^5^ http://www.ezbiocloud.net

^6^ http://ggdc.dsmz.de
