# Supplementary material for: Taxonomic identification and antagonistic activity of Streptomyces luomodiensis sp. nov. against phytopathogenic fungi
Source: Front Microbiol. 2024 May 27;15:1402653. doi: 10.3389/fmicb.2024.1402653 (PMC11163044; doi:10.3389/fmicb.2024.1402653)
Supplement: Supplementary file 1 [file Data_Sheet_1.docx]

Supplementary Material

**Taxonomic identification and antagonistic mechanism of *Streptomyces luomodiensis* sp. nov. against phytopathogenic fungi**

**Dengfeng Qi^a#^, Qiao Liu^a#^, Liangping Zou^a^, Miaoyi Zhang****^a^, Kai Li****^a^, Yankun Zhao^a^ Yufeng Chen^a^, Junting Feng^a^, Dengbo Zhou^a^, Yongzan Wei^a^, Wei Wang^a*^, Lu Zhang^b^****^*^, Jianghui Xie^a*^**

a Key Laboratory of Biology and Genetic Resources of Tropical Crops, Ministry of Agriculture, Institute of Tropical Bioscience and Biotechnology, Chinese Academy of Tropical Agricultural Sciences, Haikou, 571101, China

b Ministry of Education Key Laboratory for Ecology of Tropical Islands, Key Laboratory of Tropical Animal and Plant Ecology of Hainan Province, College of Life Sciences, Hainan Normal University, Haikou, Hainan 571158, China

#These authors have contributed equally to this work (co-first authors)

***Correspondence Authors:**

Dr. Jianghui Xie (xiejianghui@itbb.org.cn)

Dr. Lu Zhang ([luzhangtest@hainnu.edu.cn](mailto:luzhangtest@hainnu.edu.cn))

Dr. Wei Wang (wangweisys@ahau.edu.cn)

**Tables**

Table S1. Allele sequence and genome accession numbers of Streptomyces species used for the present study

| **Species** | **Strains** | **atpD** | **gyrB** | **recA** | **rpoB** | **trpB** | Genomes |
| --- | --- | --- | --- | --- | --- | --- | --- |
| SCA4-21 |  | PS467_28075 | PS467_21335 | PS467_30045 | PS467_23820 | PS467_11915 | CP117522 |
| Streptomyces iranensis | HM 35^T^ | MW197742.1 | MW197743.1 | MW197744.1 | MW197745.1 | MW197746.1 | ACEX00000000 |
| Streptomyces rapamycinicus | NRRL B-5491^T^ | M271_RS17510 | M271_RS25725 | M271_RS15490 | M271_RS22210 | M271_RS37075 | QYCY00000000 |
| Streptomyces hygroscopicus subsp. hygroscopicus | NBRC 13472 ^T^ | AXW62_RS25230 | AXW62_RS10800 | AXW62_RS21235 | AXW62_RS16240 | AXW62_RS13490 | BBOX00000000 |
| Streptomyces demainii | NRRL B-1478 (DSM41600) ^T^ | FJ406182.1 | FJ406238.1 | FJ406294.1 | FJ406349.1 | FJ406405.1 | JAURUE000000000 |
| Streptomyces melanosporofaciens | DSM 40318 ^T^ | BLV72_RS23725 | BLV72_RS16855 | BLV72_RS25665 | BLV72_RS19855 | BLV72_RS07110 | FNST00000000 |
| Streptomyces antimycoticus | NBRC 12839 ^T^ | FFT84_RS30625 | FFT84_RS23530 | FFT84_RS32640 | FFT84_RS26345 | FFT84_RS13800 | BJHV01000000 |
| Streptomyces himastatinicus | ATCC 53653 ^T^ | SSOG_RS14670 | SSOG_RS22180 | SSOG_RS12650 | SSOG_RS19135 | SSOG_RS33630 | ACEX00000000 |
| Streptomyces lydicamycinicus | NBRC 110027 ^T^ | TPA0598_01_07850 | TPA0598_02_04900 | TPA0598_08_05980 | TPA0598_05_01340 | TPA0598_12_01560 | BBNO00000000 |
| Streptomyces caniferus | NBRC 15389 ^T^ | Scani_RS04070 | Scani_RS36175 | Scani_RS02135 | Scani_RS33700 | Scani_RS26770 | BLIN00000000 |
| Streptomyces platensis | JCM 4662 (DSM 40041)^T^ | BG653_RS04710 | BG653_RS08850 | BG653_RS17255 | BG653_RS19725 | BG653_RS15125 | CP023691 |
| Streptomyces celluloflavu | NRRL B-2493 ^T^ | IH09_RS13530 | IH09_RS17885 | IH09_RS31520 | IH09_RS12580 | IH09_RS06175 | JOEL00000000 |
| Streptomyces morookaense | LMG 20074 （ DSM 40503）^T^ | HG542_RS24875 | HG542_RS03860 | HG542_RS19585 | HG542_RS18085 | HG542_RS10950 | JABBXF000000000 |
| Streptomyces lacticiproducens | GIMN4.001 （NRRL B-24800）^T^ | KT384614.1 | KT384963.1 | KT385313.1 | KT388934.1 | KT389283.1 | - |
| Streptomyces palmae | MU-AB 204 （JCM 31289）^T^ | MH534869.1 | MH534870.1 | MH534871.1 | MH534872.1 | MH534873.1 | - |
| Streptomyces cuspidosporus | NBRC 12378（NRRL B-5620）^T^ | KT384533.1 | KT384882.1 | KT385230.1 | KT388852.1 | KT389202.1 | - |
| Streptomyces sparsogenes | ATCC 25498 ^T^ | BBH68_RS20645 | BBH68_RS13335 | BBH68_RS22740 | BBH68_RS16075 | BBH68_RS02210 | MAXF01000000 |

Table S2. Physiological and biochemical characteristics of strain SCA4-21^T^

| **Characteristics** | **Result** | **Characteristics** | **Result** |
| --- | --- | --- | --- |
| **Morphological** |  | **Antibiotic sensitivity (μg/slice)** |  |
| Aerial mycelium | Branched | Clindamycin (2) | S |
| Spore chain shape | Spiral | Chloramphenicol (30) | S |
| Spore shape | Cylindrical | Furazolidone (300) | R |
| Spore surface | Shrinkage | Compound sulfamethoxazole (1.25) | R |
| **Biochemical** |  | Polymyxin B (300 IU/slice) | R |
| Urease test | + | Vancomycin (30) | R |
| Twain 20 | + | Penicillin (10 U/slice) | S |
| Twain 40 | - | Erythromycin (15) | R |
| Twain 60 | - | Minocycline (30) | R |
| Gelatin liquefaction | - | Kanamycin (30) | R |
| Starch hydrolysis | + | Gentamicin (10) | R |
| Nitrate reduction | - | Cefepime (75) | S |
| Degradation of cellulose | - | Ceftriaxone (30) | R |
| Melanoid pigment | + | Cefotaxime (30) | S |
| H_2_S production | - | Cefuroxime (30) | R |
| Siderophores | + | Cefamandole (30) | S |
| **Physiological** |  | Midecamycin (30) | S |
| pH range for growth | 6-9 | Carbenicillin (100) | S |
| Optimum pH for growth | 7 | Ampicillin (10) | S |
| NaCl tolerance (%) | 1-3 | Benzylpenicillin (1) | S |
| Optimum NaCl toleranc (%) | 2.00 | Piperacillin (100) | S |

Note: Note: “+” means positive; “-” means negative; S, sensitivity; R, resistance.

Table S3. The carbon and nitrogen utilization of strain SCA4-21T

| **Characteristic** | **Results** |
| --- | --- |
| *Carbon source utilization* |  |
| D- mannose | +++ |
| D- trehalose | ++ |
| L- arabinose | - |
| Sorbose | +++ |
| D- fructose | ++ |
| α- Lactose | + |
| D- ribose | +++ |
| Mannitol | ++ |
| Xylan | + |
| Raffinose | - |
| Inositol | - |
| *Nitrogen source utilization* |  |
| L- phenylalanine | + |
| L- asparagine | ++ |
| Anhydrous inositol | - |
| L- methionine | + |
| L- valine | +++ |
| L- histidine | + |
| L- glutamic acid | - |
| L- tryptophan | ++ |
| L- tyrosine | - |
| D- cellobiose | +++ |
| L- arginine | - |
| L- hydroxyproline | + |
| L- cysteine | + |
| Glycine | - |

Note: “+++” represented that the strain grew well; “+ +” represented the general growth of the strain; “+” represented that the strain can grow; ‘-’ represented the strain cannot grow.

Table S4. COG functional cluster of predicted genes in strain SCA4-21T

| **COG functional categories** | **Type** | **Gene**  **No.** | **% of Gene**  **No**. |
| --- | --- | --- | --- |
| **Information storage and processing** |  | **1489** | **19.7%** |
| RNA processing and modification | A | 1 |  |
| Translation, ribosomal structure and biogenesis | J | 296 |  |
| Transcription | K | 1032 |  |
| Replication, recombination and repair | L | 160 |  |
| **Metabolism** |  | **3578** | **47.4%** |
| Energy production and conversion | C | 441 |  |
| Amino acid transport and metabolism | E | 589 |  |
| Nucleotide transport and metabolism | F | 138 |  |
| Carbohydrate transport and metabolism | G | 772 |  |
| Coenzyme transport and metabolism | H | 484 |  |
| Lipid transport and metabolism | I | 568 |  |
| Inorganic ion transport and metabolism | P | 309 |  |
| Secondary metabolites biosynthesis, transport and  catabolism | Q | 277 |  |
| **Cellular processes and signaling** |  | **1528** | **20.2%** |
| Cell cycle control, cell division, chromosome  partitioning | D | 86 |  |
| Cell wall/membrane/envelope biogenesis | M | 310 |  |
| Cell motility | N | 20 |  |
| Posttranslational modification, protein turnover,  chaperones | O | 255 |  |
| Signal transduction mechanisms | T | 484 |  |
| Intracellular trafficking, secretion, and vesicular  transport | U | 53 |  |
| Defense mechanisms | V | 212 |  |
| Extracellular structures | W | 14 |  |
| Cytoskeleton | Z | 5 |  |
| Mobilome: prophages, transposons | X | 89 |  |
| **Poorly characterized** |  | **953** | **12.6%** |
| General function prediction only | R | 794 |  |
| Function unknown | S | 159 |  |

Table S5. Family and gene ID of strain SCA4-21^T^ CAZymes

| **Family** | **Genes No.** | **Gene ID** |
| --- | --- | --- |
| **Auxiliary Activities（AA）** | **36** |  |
| AA1 | 2 | gene0968, gene2542 |
| AA2 | 1 | gene6975 |
| AA3 | 11 | gene0094, gene0190, gene1355, gene1407, gene1519, gene2924, gene3679, gene3692, gene4873, gene6812, gene7011 |
| AA3_2 | 3 | gene1526, gene3647, gene6518 |
| AA4 | 3 | gene3323, gene7037, gene7935 |
| AA5 | 1 | gene3264 |
| AA6 | 5 | gene0059, gene0636, gene7628, gene7709, gene8112 |
| AA7 | 3 | gene1000, gene4505, gene6047 |
| AA10 | 7 | gene0624, gene1675, gene1780, gene2049, gene3257, gene5523, gene6167 |
| **Carbohydrate-Binding Modules (CBM)** | **6** |  |
| CBM2 | 1 | gene2825 |
| CBM13 | 2 | gene5170, gene7842 |
| CBM35 | 1 | gene3777 |
| CBM66 | 2 | gene6392, gene7306 |
| **Carbohydrate Esterases (CE)** | **74** |  |
| CE1 | 24 | gene0176, gene0177, gene0396, gene0570, gene0767, gene1439, gene1563, gene1656, gene1898, gene2671, gene3543, gene3567, gene3714, gene3914, gene4651, gene4833, gene5174, gene5350, gene5357, gene7311, gene7724, gene7864, gene7996, gene8217 |
| CE2 | 2 | gene0498, gene0681 |
| CE3 | 6 | gene1102, gene2034, gene3277, gene3491, gene6265, gene6586 |
| CE4 | 7 | gene2216, gene4634, gene4840, gene6952, gene7425, gene7656, gene8060 |
| CE7 | 5 | gene0562, gene4053, gene7026, gene7877, gene8194 |
| CE8 | 1 | gene0661 |
| CE9 | 1 | gene3774 |
| CE10 | 21 | gene0003, gene0081, gene0199, gene0305, gene0476, gene1047, gene1138, gene1175, gene1685, gene1712, gene3597, gene4022, gene4408, gene5315, gene5675, gene6198, gene6272, gene6316, gene6361, gene7673, gene7840 |
| CE12 | 2 | gene2218, gene7487 |
| CE14 | 4 | gene1650, gene5187, gene5317, gene6111 |
| CE15 | 1 | gene7746 |
| **Glycoside Hydrolases (GH)** | **155** |  |
| GH1 | 2 | gene1587, gene3177 |
| GH2 | 8 | gene0100, gene0974, gene6895, gene6996, gene7224, gene7387, gene8209, gene8222 |
| GH3 | 4 | gene3119, gene5445, gene6959, gene8067 |
| GH4 | 3 | gene3020, gene6988, gene0953 |
| GH5_1 | 1 | gene3947 |
| GH5_8 | 1 | gene7006 |
| GH5_18 | 1 | gene8068 |
| GH5_19 | 1 | gene6958 |
| GH5_40 | 1 | gene3171 |
| GH5_43 | 1 | gene0548 |
| GH6 | 7 | gene0172, gene1998, gene3172, gene3265, gene3949, gene5764, gene6217 |
| GH8 | 1 | gene3962 |
| GH9 | 1 | gene7050 |
| GH10 | 2 | gene1734, gene6953 |
| GH11 | 1 | gene4355 |
| GH12 | 1 | gene5893 |
| GH13_3 | 1 | gene5658 |
| GH13_9 | 1 | gene5655 |
| GH13_10 | 1 | gene6165 |
| GH13_11 | 2 | gene5690, gene6169 |
| GH13_13 | 1 | gene2526 |
| GH13_16 | 1 | gene5657 |
| GH13_26 | 1 | gene6168 |
| GH13_30 | 2 | gene2532, gene7053 |
| GH13_32 | 1 | gene2527 |
| GH15 | 3 | gene2098, gene2175, gene7351 |
| GH16 | 4 | gene0917, gene5654, gene6893, gene7651 |
| GH18 | 10 | gene1565, gene1638, gene2872, gene2907, gene3179, gene5217, gene5218, gene5259, gene5648, gene6873 |
| GH19 | 1 | gene6597 |
| GH20 | 3 | gene3148, gene3345, gene3624 |
| GH23 | 3 | gene1828, gene2670, gene4503 |
| GH25 | 3 | gene4119, gene5503, gene5697 |
| GH26 | 1 | gene0108 |
| GH27 | 1 | gene0179 |
| GH30_5 | 2 | gene4557, gene4926 |
| GH30_7 | 1 | gene2753 |
| GH31 | 2 | gene1301, gene3371 |
| GH33 | 4 | gene1036, gene2559, gene6377, gene7662 |
| GH35 | 1 | gene7807 |
| GH36 | 3 | gene0101, gene0376, gene6585 |
| GH42 | 1 | gene5892 |
| GH43 | 1 | gene1515 |
| GH43_3 | 1 | gene7226 |
| GH43_5 | 1 | gene0563 |
| GH43_10 | 1 | gene7070 |
| GH43_24 | 1 | gene0553 |
| GH43_26 | 2 | gene0554, gene6994 |
| GH43_34 | 2 | gene0507, gene0555 |
| GH44 | 1 | gene7198 |
| GH46 | 1 | gene6600 |
| GH48 | 1 | gene3170 |
| GH51 | 3 | gene2748, gene6815, gene6831 |
| GH54 | 1 | gene0178 |
| GH55 | 1 | gene0843 |
| GH62 | 2 | gene0173, gene1735 |
| GH63 | 1 | gene2853 |
| GH64 | 2 | gene2099, gene7246 |
| GH65 | 2 | gene0539, gene0972 |
| GH67 | 1 | gene0266 |
| GH74 | 1 | gene1467 |
| GH76 | 2 | gene6995, gene7247 |
| GH77 | 1 | gene3010 |
| GH78 | 2 | gene0515, gene2612 |
| GH85 | 1 | gene8066 |
| GH87 | 4 | gene1641, gene7052, gene7828, gene7829 |
| GH89 | 1 | gene2524 |
| GH92 | 4 | gene0931, gene6109, gene6112, gene7344 |
| GH93 | 3 | gene2646, gene7412, gene8210 |
| GH95 | 1 | gene0583 |
| GH106 | 2 | gene0514, gene0516 |
| GH109 | 4 | gene0602, gene2576 |
| GH113 | 1 | gene7311 |
| GH114 | 2 | gene2359, gene6983 |
| GH127 | 2 | gene0557, gene8140 |
| GH128 | 1 | gene7213 |
| GH135 | 1 | gene5362 |
| GH145 | 1 | gene6910 |
| GH146 | 3 | gene0517, gene0552, gene8139 |
| GH152 | 1 | gene7212 |
| GH154 | 2 | gene1022, gene7690 |
| **Glycosyl Transferases (GT)** | **65** |  |
| GT1 | 5 | gene0005, gene1180, gene3194, gene3883, gene6414 |
| GT2_Glycos_transf_2 | 12 | gene0098, gene1210, gene1211, gene3460, gene3581, gene3863, gene4092, gene6148, gene6560, gene6827, gene6879, gene7922 |
| GT2_Glyco_tranf_2_3 | 11 | gene0027, gene0030, gene1219, gene1383, gene1997, gene2580, gene3613, gene3952, gene6830, gene6852, gene7308 |
| GT4 | 15 | gene0925, gene1215, gene1216, gene1333, gene1334, gene1987, gene1996, gene2081, Gene2100, gene2448, gene2614, gene3874, gene6502, gene6711, gene7874 |
| GT5 | 1 | gene1807 |
| GT9 | 3 | gene1212, gene1217, gene6836 |
| GT20 | 1 | gene3769 |
| GT28 | 3 | gene2400, gene5130, gene7422 |
| GT35 | 1 | gene5662 |
| GT39 | 1 | gene5075 |
| GT41 | 2 | gene2299, gene3007 |
| GT76 | 2 | gene2068, gene4393 |
| GT81 | 1 | gene3768 |
| GT83 | 4 | gene0158, gene3953, gene4091, gene7362 |
| GT87 | 3 | gene2632, gene4908, gene7459 |
| **Polysaccharide Lyases (PL)** | **12** |  |
| PL1 | 1 | gene7071 |
| PL1_5 | 1 | gene0392 |
| PL1_6 | 1 | gene2217 |
| PL3_4 | 1 | gene5660 |
| PL7_3 | 2 | gene7568, gene7569 |
| PL8 | 1 | gene7236 |
| PL9 | 1 | gene6336 |
| PL9_3 | 1 | gene2215 |
| PL11 | 1 | gene5661 |
| PL26 | 1 | gene6698 |
| PL31 | 1 | gene2302 |
| **Total No.** | **348** |  |

Figures


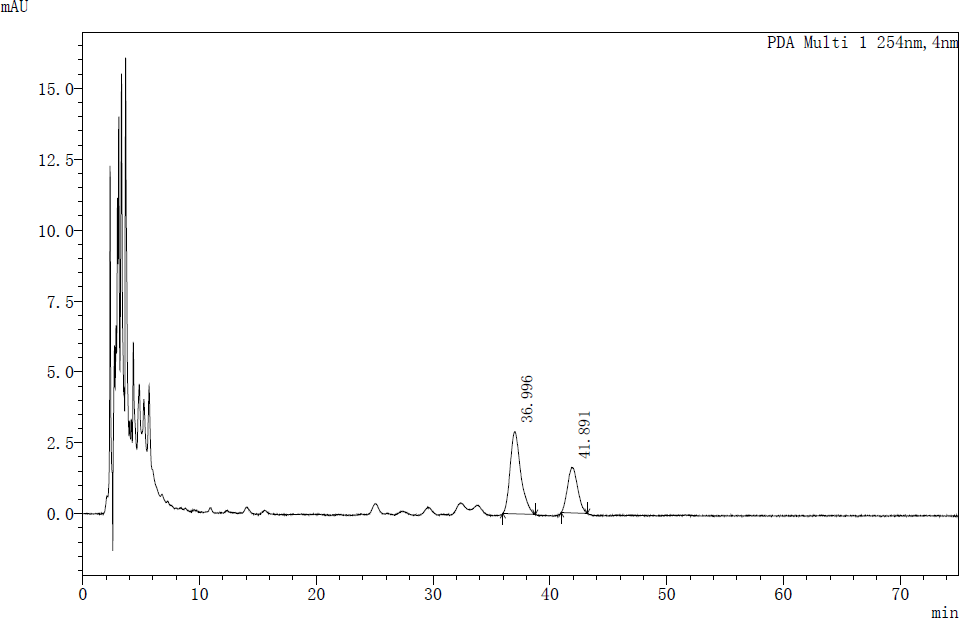


Fig. S1. The analysis of high-performance liquid chromatography (Shimadzu LabSolutions) showed that strain SCA4-21T mainly contained dominant menaquinones composed of MK9 (H8) (65.50%) and MK10 (H2) (34.50%).

**
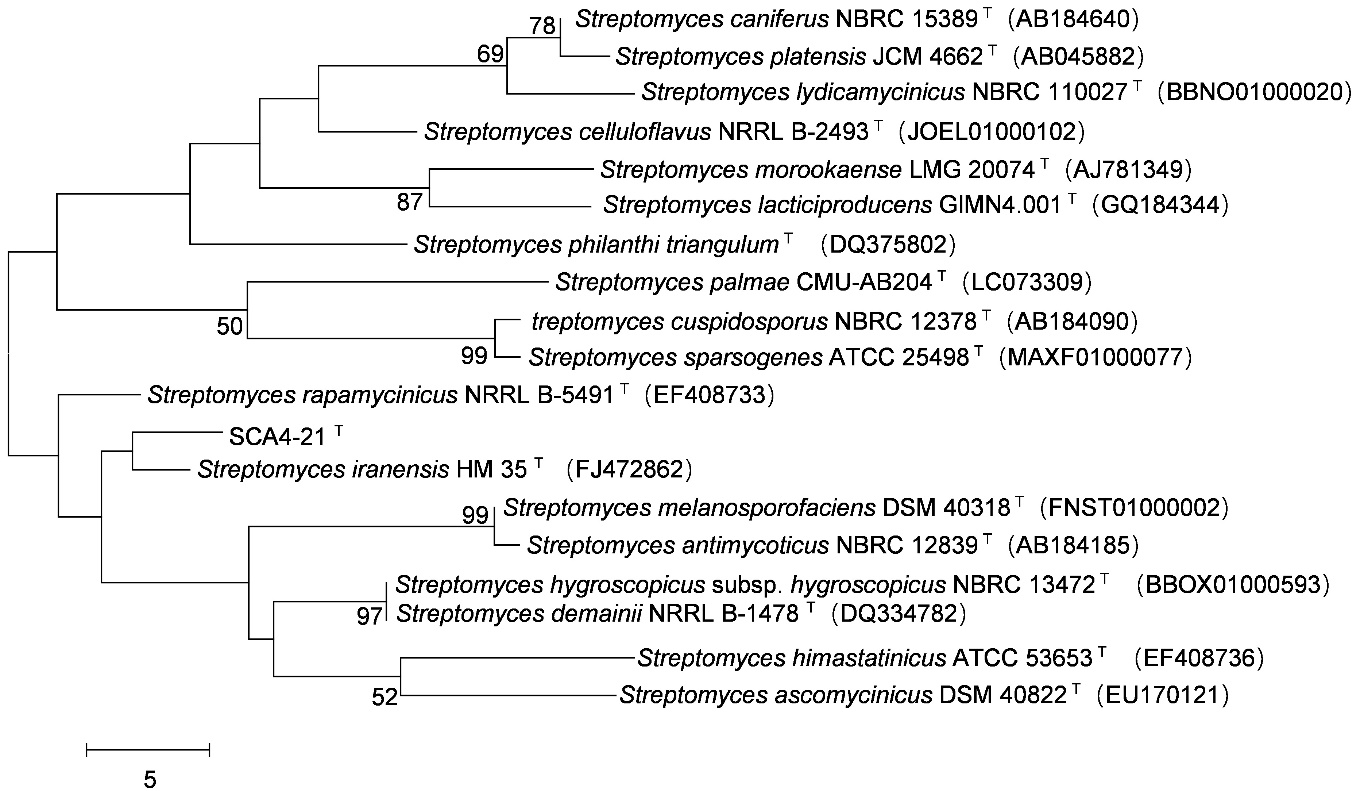
**

Fig. S2. Maximum-parsimony tree based on 16S rRNA gene showing position of strain SCA4-21T among its phylogenetic neighbors in the genus *Streptomyces*. Bootstrap percentages (based on 1000 replications) were shown at branching points. Bar, 5 base changes between nodes.


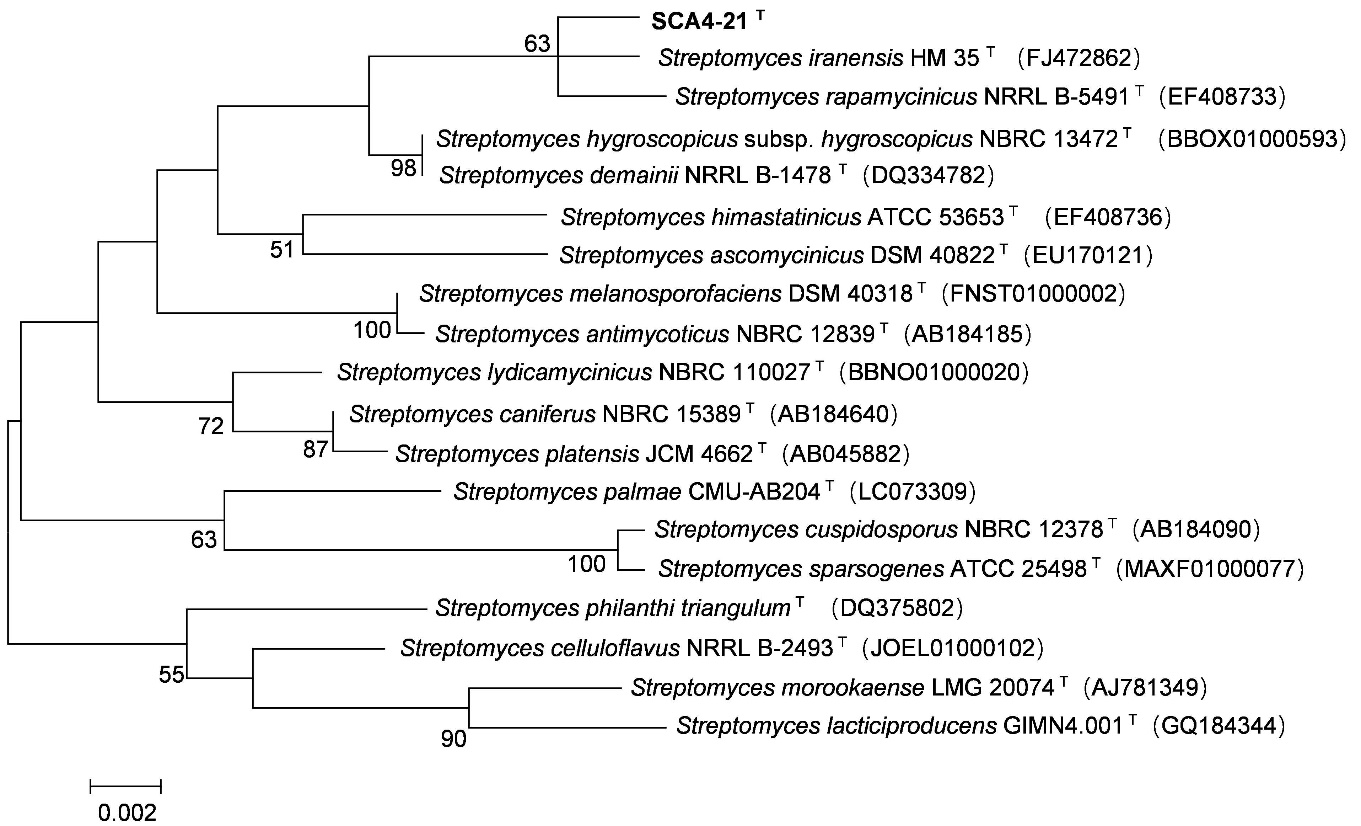


Fig. S3. Maximum-likelihood tree based on 16S rRNA gene showing position of strain SCA4- 21T among its phylogenetic neighbors in the genus *Streptomyces*. Bootstrap percentages (based on 1000 replications) were shown at branching points. Bar, 0.002 substitutions per nucleotide position.


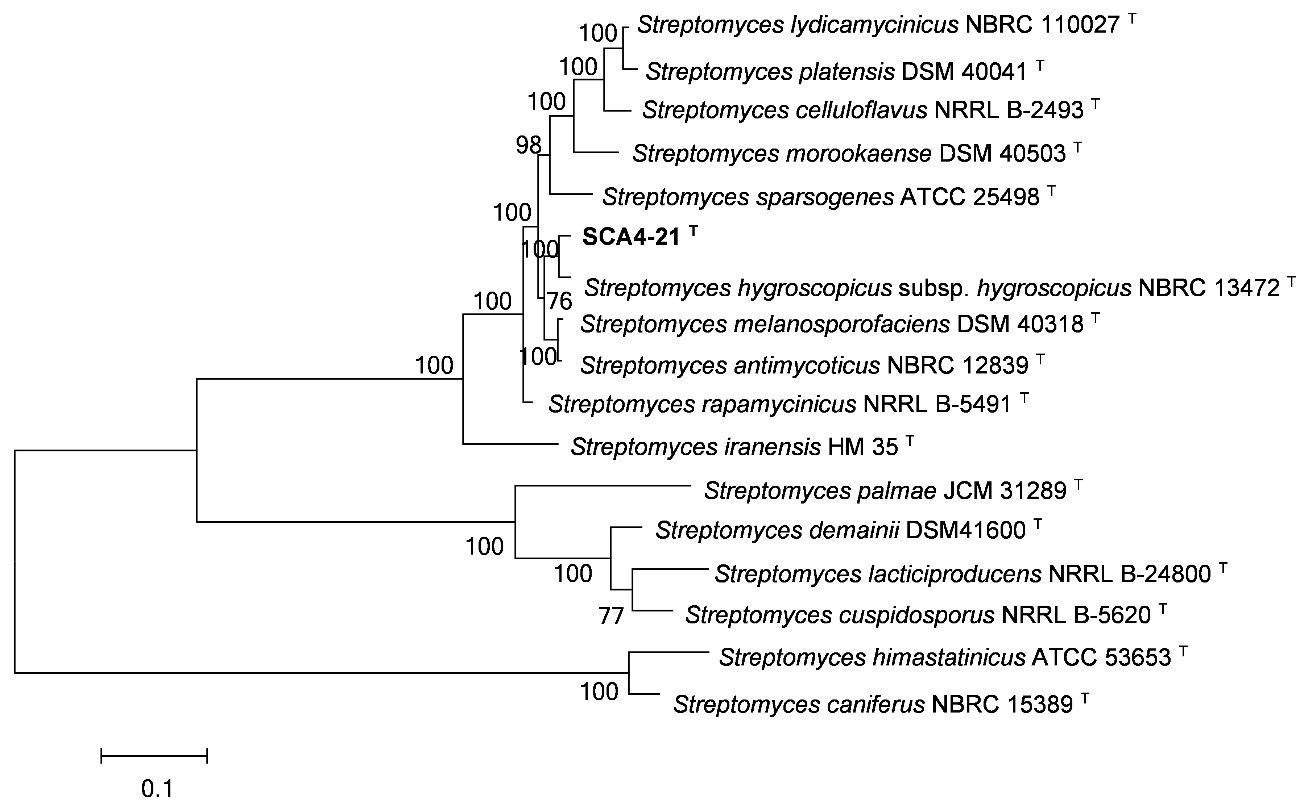


Fig. S4. Maximum-likelihood tree based on five housekeeping genes (*atpD*, *gyrB*, *recA*, *rpoB*, and *trpB*) showing position of strain SCA4-21T among its phylogenetic neighbors in the genus *Streptomyces*. Bootstrap percentages (based on 1000 replications) were shown at branching points. Bar, 0.1 substitutions per nucleotide position.


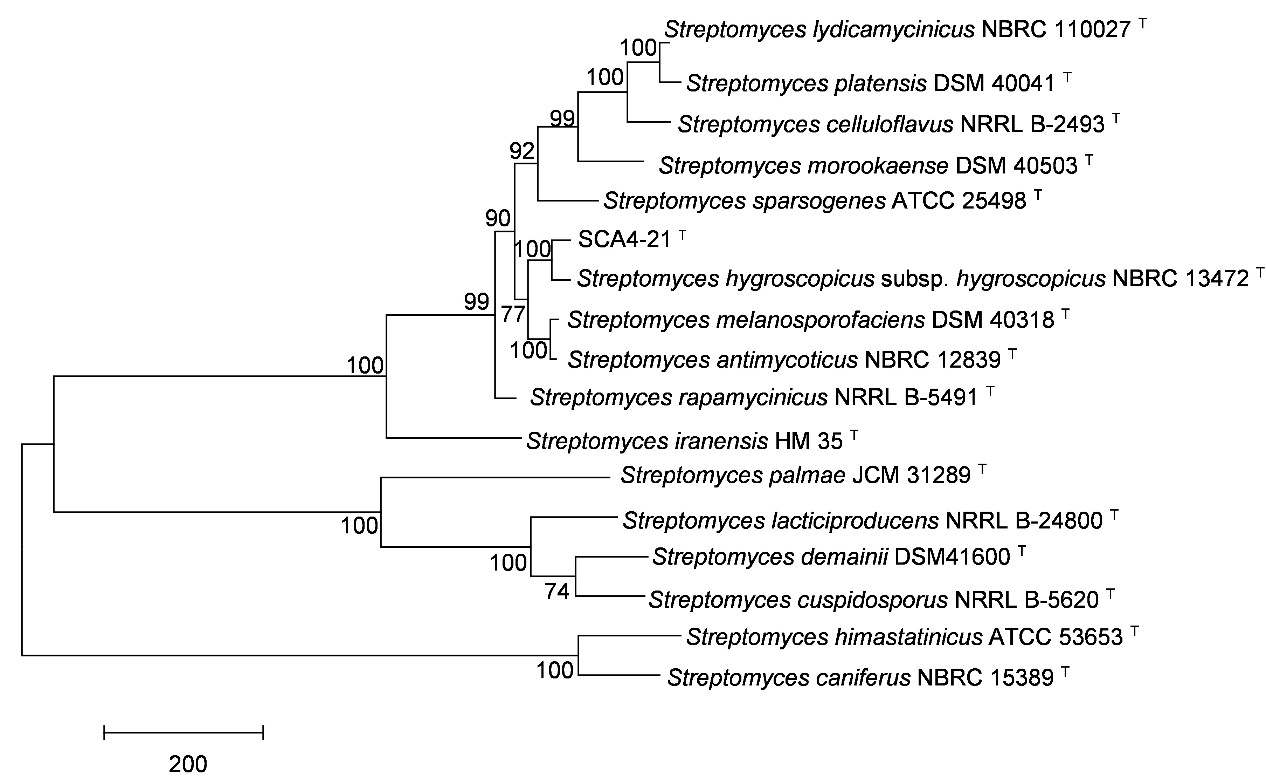


Fig. S5. Maximum-parsimony tree based on five housekeeping genes (*atpD*, *gyrB*, *recA*, *rpoB*, and *trpB*) showing position of strain SCA4-21T among its phylogenetic neighbors in the genus *Streptomyces*. Bootstrap percentages (based on 1000 replications) were shown at branching points. Bar, Bar, 200 base changes between nodes.

**
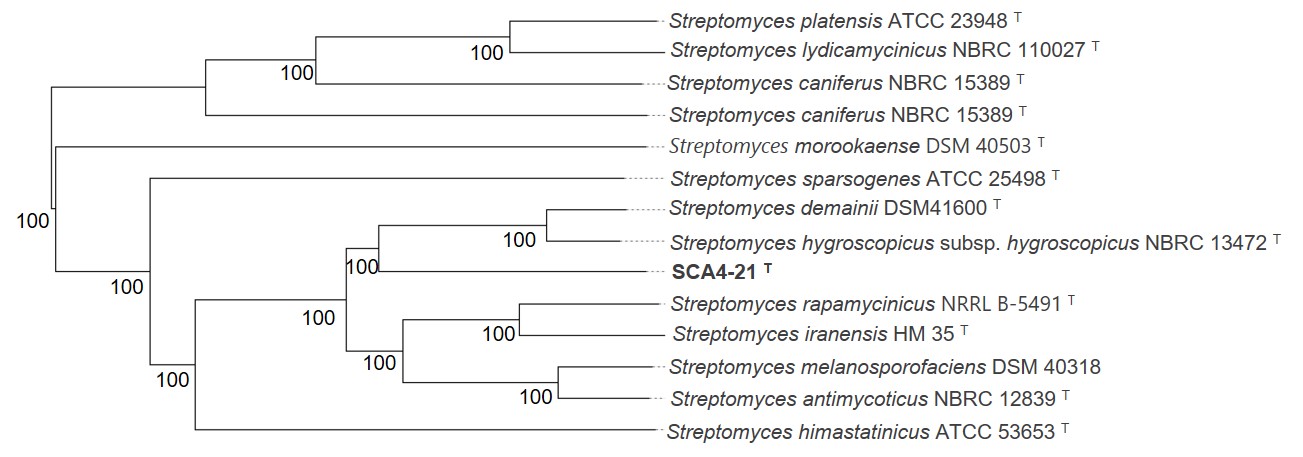
**

Fig. S6.  A phylogenomic tree of stain SCA4-21 ^T^ and related *Streptomyces* species. The accession numbers of 14 type strain genomes were uploaded to TYGS server. Tree inferred with FastME 2.1.6.1 (Lefort et al., 2015) from the Genome BLAST Distance Phylogeny (GBDP) calculated from genome sequences. The branch lengths are scaled in terms of GBDP distance formula d5 (Meier-Kolthoff et al., 2015). The numbers above branches are GBDP pseudo-bootstrap support values > 60 % from 100 replications, with an average branch support of 100.0 %. The tree was rooted at the midpoint (Farris, 1972).

References

Farris, J. S. (1972). Estimating phylogenetic trees from distance matrices. *Am Nat*. 106: 645–667.

Lefort, V., Desper, R., and Gascuel, O. (2015). FastME 2.0: A comprehensive, accurate, and fast distance-based phylogenyinference program. *Mol Biol Evol*. 32: 2798–2800. doi: 10.1093/molbev/msv150

Meier-Kolthoff, J. P., Auch, A. F., Klenk, H-P., and Göker, M. (2013). Genome sequence-based species delimitation with confidence intervals and improved distance functions. *BMC Bioinformatics*. 14: 60. doi: 10.1186/1471-2105-14-60
